# A comparative study of moisture adsorption on GO, MOF-5, and GO/MOF-5 composite for applications in atmospheric water harvesting†

**DOI:** 10.1039/d4na00150h

**Published:** 2024-06-07

**Authors:** Muhammad Saeed-Ul-Hassan, Muhammad Ehtisham, Ahmad K. Badawi, Asad Muhammad Khan, Rafaqat Ali Khan, Bushra Ismail

**Affiliations:** a Department of Chemistry, COMSATS University Islamabad Abbottabad Campus-22060 Pakistan msaeed5651123@gmail.com muhammadehtasham10@gmail.com amkhan@cuiatd.edu.pk rafaqatali@cuiatd.edu.pk bushraismail@cuiatd.edu.pk +92 992 383595 +92 992 383592; b Civil Engineering Department, El-Madina Higher Institute for Engineering and Technology Giza 12588 Egypt dr.ahmedkaram91@gmail.com

## Abstract

Water scarcity is an alarming situation across the globe. Several methods have been reported in the literature to minimize the water shortage problem. Sorbent-based atmospheric water harvesting (SBAWH) is considered an energy-efficient, low-cost strategy, and sustainable approach. In the present study, the synthesis of graphene oxide (GO) was carried out using a modified Hummers' method, while the synthesis of MOF-5 and a GO/MOF-5 composite was carried out using a solvothermal approach. The synthesized materials were characterized by X-ray diffraction analysis (XRD), scanning electron microscopy (SEM), and Fourier transform infrared spectroscopy (FTIR). The phase composition and crystallinity of all synthesized samples were confirmed by XRD analysis. SEM analysis provided information about the surface morphology of all synthesized samples. The adsorption of water vapors on surfaces of GO, MOF-5, and the GO/MOF-5 composite was evaluated by FTIR analysis. The negative charge was explored by the PZC technique on the surface of all synthesized materials. The water adsorption characteristics of GO, MOF-5, and the GO/MOF-5 composite were evaluated using an atmospheric water harvesting (AWH) plant. The maximum adsorption capacity of 542 mg g^−1^ was achieved by the MOF at 55% RH (relative humidity), while a low adsorption capacity of the MOF was observed at higher humidity values. This problem was overcome by making a GO/MOF-5 composite. GO imparts structural stability to the MOF-5 structure at higher humidity values. The maximum adsorption capacity of 1137 mg g^−1^ was achieved by the GO/MOF-5 composite at 75% RH. Several isotherm models, such as Langmuir, Freundlich, and Temkin, were applied to confirm the single-site occupation by water molecules and chemisorption behavior. Several thermodynamic properties were calculated, including isosteric heat (*Q*_st_), Gibbs free energy (Δ*G*), and sorption entropy (Δ*S*). The overall thermodynamics study confirms that the adsorption process is spontaneous and exothermic. In addition, second-order kinetics confirms that all synthesized material shows chemisorption behavior.

## Abstract 2

Water scarcity is an alarming situation across the globe.

## Graphical abstract

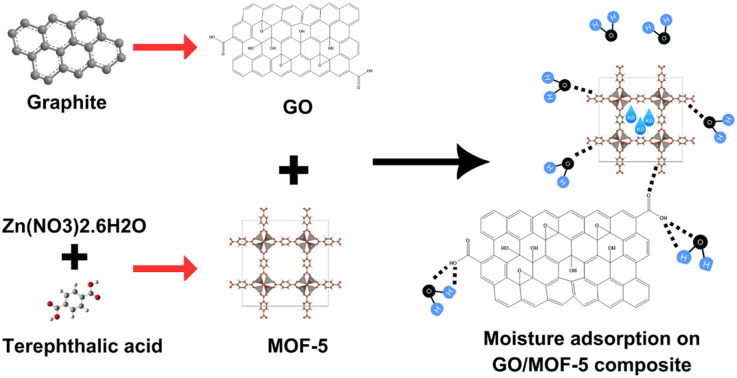

## 1. Introduction

Presently, shortages of water are now recognized as a prospective global issue. Although water covers 70% of the Earth's surface, only 2.5% of it is accessible as freshwater.^1^ The lack of water poses a significant challenge to the progress and advancement of human development. Statistics indicate that over two-thirds of the world's population, which amounts to 4 billion individuals, experiences acute water scarcity for a minimum of one month annually. Additionally, 500 million people endure persistent water shortages throughout the whole year.^2^ Groundwater in arid regions is persistently diminishing, and the water quality may pose risks to human well-being.^3^ Even non-arid regions with moderate yearly rainfall are also experiencing water stress.^4^ Power generation accounts for the largest share of global water consumption, and there might soon be a situation between prioritizing drinking water and meeting energy needs.^5^ Over fifty percent of the world's population will face water stress by 2050 as a result of rising populations, excessive resource usage, industrialization, and climate change.^6^ Hence, humans must discover a means of acquiring potable water in desert regions.

The atmosphere contains over 13 sextillions (10^21^) liters of water, in the form of water vapors or droplets. This accounts for roughly 10% of all freshwater resources. The presence of water in our environment is a highly significant renewable resource, and the implementation of atmospheric water harvesting (AWH) technology is anticipated to reduce the impact of water scarcity on human beings.^7^

Various atmospheric water harvesting (AWH) techniques have been used in previous studies.^8,9^ The mechanism of SBAWH involves water vapor adsorption from air and its conversion into liquid water. There are three different approaches used in the AWH process: (1) fog capture using nets;^10^ (2) the condensation process;^11^ and (3) sorbent-based atmospheric water harvesting (SBAWH).^12^ The process of fog capture is restricted to specific geographical areas. In the condensation process, the ambient air must be cooled below its dew point temperature, and it requires a large amount of energy.^13^

SBAWH has received great attention from researchers due to its cost effectiveness and capability of functioning well in arid environments. The ideal adsorbents should possess high water uptake, low energy demand for water release, fast water capture/release, high cycling stability, and low cost.^14^ Numerous adsorbent materials are employed in SBAWH, including silica gel, hygroscopic substances, hydrogels, zeolite, and metal–organic framework (MOF). Conventional adsorbents are ideal for moisture capture, but their high regeneration temperature and low adsorption capacity compared to MOFs limit their application in AWH.^15^

MOF nanocomposites can be utilized in several applications, such as catalysis, wastewater treatment, drug delivery, and adsorption. MOFs act as suitable adsorbents due to several properties, such as high porosity and high adsorption capacity, which makes them ideal for SBAWH.^16^ A group of researchers used MOF-801 as an effective adsorbent for moisture capture, achieving an adsorption capacity of 1 L kg^−1^ day^−1^.^17^ Numerous adsorbents have been previously used in the literature, such as Co-based MOF-31,^18^ MOF-801 (Zr_6_O_4_(OH)_4_(fumarate)_6_), MOF-303 (Al(OH)(HPDC)),^19^ poly(2-acrylamido-2-methyl-1-propanesulfonic acid) (PAMPS) hydrogel,^20^ porous carbon cuboids,^21^ and Zn_1−*x*_Co_*x*_Fe_2_O_4_ (*x* = 0.6).^22^

In the present study, the water vapor adsorption capacity of GO, MOF-5, and a GO/MOF-5 composite was compared. MOF-5 is considered a good adsorbent due to its porous structure. Graphene oxide (GO) is composed of several functional groups, including COOH, OH, and RCOR. GO's groups and layer structure make it suitable for various adsorption applications. When combined with MOF-5, the oxygen-containing functional groups in GO can react with the metal ions in MOF-5 to create a GO/MOF-5 composite, while GO imparts extra stability to the MOF-5 structure. The objective of this study was to prepare and characterize the GO/MOF-5 composite and use it as a sorbent for moisture adsorption studies for application in atmospheric water harvesting.

## 2. Experimental

### 2.1 Synthesis of graphene oxide

To produce the graphene oxide, a modified Hummer's process was employed. Graphite powder (1 g), sodium nitrate (0.5 g), and sulfuric acid (25 mL) were mixed, and the resultant solution was sterilized under a cold bath for thirty minutes. After stirring, 3 g of KMnO_4_ was progressively added, and the mixture was then stirred for a further 3 hours. The solution was stirred for an extra hour at 35 °C after being removed from the cold bath. Finally, 50 mL of water was poured and stirred for an hour and further sterilized for another hour with the addition of 100 mL of deionized water. To remove any excess potassium permanganate, 5 mL of hydrogen peroxide was added. Afterward, the precipitate was separated by centrifugation, washed with distilled water to remove unreacted residue, and then dried inside the vacuum oven at 80 °C.^23^

### 2.2 Synthesis of MOF-5

MOF-5 was synthesized by a previously reported procedure. First, 0.62 g of zinc nitrate hexahydrate and 0.16 g of terephthalic acid were completely dissolved in 20 mL of dimethyl formamide (DMF). Subsequently, 11 mL of tetra ethylene amine (TEA) was gradually added while stirring. After that, the resulting mixture was agitated for two hours at ambient temperature. Following the completion of the reaction, the solid-form precipitates were recovered by centrifugation, and they were then added to DMF for three days and washed three times with ethanol, respectively. Finally, the powder MOF-5 was dried at 80 °C for 12 hours.^24^

### 2.3 Synthesis of composite

**Fig. 1:**
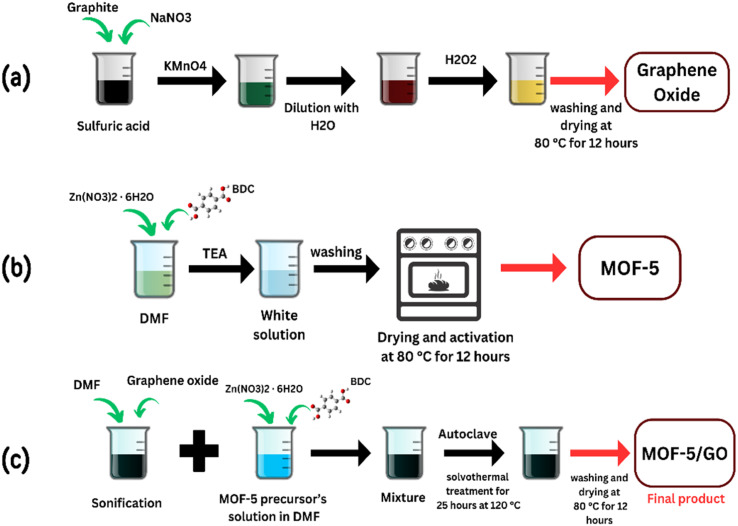
Scheme for the synthesis of (a) GO, (b) MOF-5, and (c) GO/MOF-5.

To create a DMF emulsion of GO, 0.33 g of GO was sonicated and dispersed in DMF. The solvothermal process was used to synthesize the GO/MOF-5 composite with some modifications. In short, Zn (NO_3_)_2_·6H_2_O (5.2 g) and 1,4-benzene dicarboxylic acid (BDC) (1.0 g) were dissolved in 35 mL of GO/DMF solution. The resulting mixture was treated solvothermally at 120 °C for 25 hours. Finally, the obtained sample was washed with DMF and ethanol, and the GO/MOF-5 composite was produced by vacuum drying at 80 °C for 12 hours (Fig. 1).^25^

### 2.4 Characterization

The XRD analysis, conducted using the PAN analytical 3040/60X'Pert PRO, was used to assess the composition of the phases and crystallinity. The lattice parameter (*a*), volume of cell (*V*_cell_), crystallite size (*D*), and X-ray density (*ρ*_x-ray_) of the synthesized samples were determined by applying the following equations.

1 *a* = *d*^2^ (*h*^2^ + *k*^2^+ *l*^2^)^1/2^

where “*d*” is the value of the *d*-spacing of lines in the XRD pattern, and “*hkl*” is related to Miller indices.

2 *V*_cell_ = *a*^3^

Here, “*V*_cell_” represents unit cell dimensions, and “*a*” is the crystal lattice parameters.3
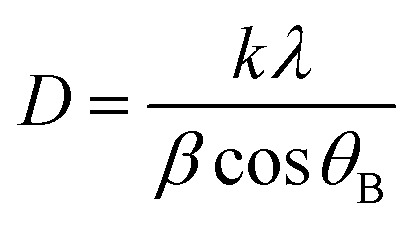
“*D*” represents Scherrer crystallite size, “*k*” represents the shape factor, and it depends on the composition of the crystal. *β* is the broadening of the diffraction peak measured at half the width of its ultimate intensity, *λ* is the X-ray wavelength and is equal to 1.542 A°, and *θ*_B_ is Bragg's angle.4
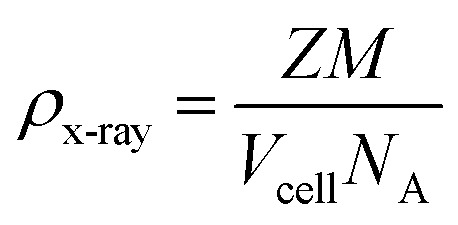
“*Z*” is the number of molecules per formula unit, *M* is the molar mass, “*N*_A_” represents Avogadro's number (6.022 ×10^23^), and “*ρ*” denotes the X-ray density.

### 2.5 Point of zero charge

The point of zero charge was determined using the salt addition method. In the salt addition method, 12.5 mg of the sample was mixed in 5 mL of NaNO_3_ solution having a concentration of 0.1 M while keeping the pH between 2, 4, 6, 8, 10, and 12. Dropwise additions of 0.1 M solutions of HCl and NH_4_OH were used to alter the initial pH (pH_i_) readings. All these samples were put on an orbital shaker for 24 hours to check for pH changes after maintaining the appropriate pH levels. The pH_pzc_ value may be found by plotting a graph between the change in pH (ΔpH) and the initial pH (pH_i_). When the value of pH is higher than pH_pzc_ (pH > pH_pzc_), the surface is negatively charged, and when it is smaller than pH_pzc_ (pH < pH_pzc_), it contains a positive charge.^26^

## 3. Adsorption studies

The freshly prepared samples were washed, filtered, and dried at 80 °C for two hours to achieve a constant weight. The water vapor adsorption was carried out using the AWH plant. An AWH plant was simply made up of glass in which a humidifier was placed to generate water vapors, and a small fan was used to spread out water vapors in a closed environment. Furthermore, a small screen was employed to check the change in humidity and temperature inside the closed AWH plant.

The adsorption experiments were carried out by placing some freshly prepared samples in a closed system and checking their moisture content by varying the RH from 45% to 95%. The material's overall moisture content was calculated using gravimetric analysis. Initially, the dry weight of the sample was measured and then placed in an AWH plant for 20 minutes. The overall weight changes before and after water vapor adsorption give information about the moisture content in each sample. The same process was repeated again and again until a saturation point was achieved. The highest amount of water captured at saturation point was the equilibrium moisture content (EMC) of all the samples. The same process was repeated to get the moisture content values for all samples in various humidity conditions.

### 3.1 Isotherm modeling

To find out the adsorption capacity of each sample, the moisture content plotted against time, which yields the value of equilibrium moisture content, is done using experimental data from the water vapor adsorption experiment described above. The amount of water in the material is indicated by its moisture content (Mc). The formula below can be used to determine moisture content.5
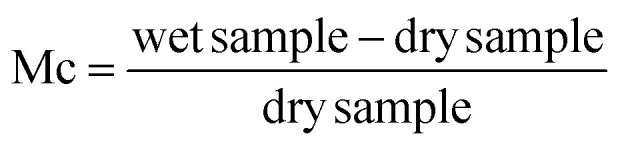


The measured moisture content can be expressed as (g g^−1^) or (mg g^−1^). The plot of the moisture content at equilibrium and the activity of water (*a*_w_) may also be explored using the Mc values.

The adsorption isotherm is a significant factor in the process of adsorption. The adsorption isotherms describe the underlying events and interactions that occur between the adsorbate and adsorbent. In general, the predictive capability of adsorption performance can be attributed to the utilization of adsorption isotherm modeling. In this study, we used several models, such as Langmuir, Freundlich, and Temkin models, to evaluate the adsorption process. Every model has its significance.

The following equation represents the Langmuir isotherm.^27^6
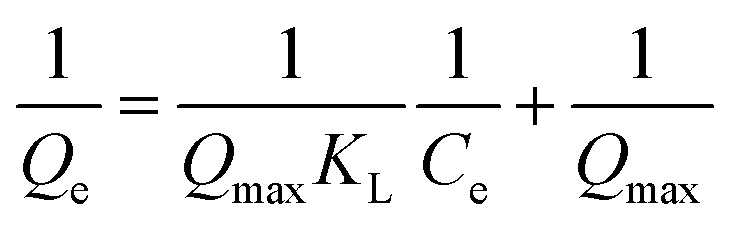
7
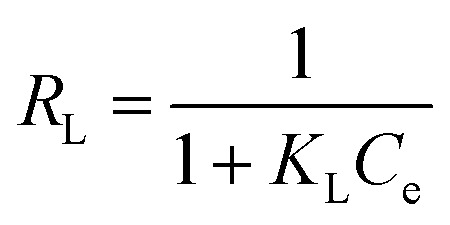
where *Q*_max_ is the adsorbent monolayer capacity (mg g^−1^), *Q*_e_ is the amount of absorbed adsorbate molecule per gram of absorbent (mg g^−1^), *C*_e_ is the adsorbate equilibrium concentration (mg g^−1^), and *K*_L_ is the Langmuir adsorption constant.

The following equation is used to represent the Freundlich isotherm:^27^8
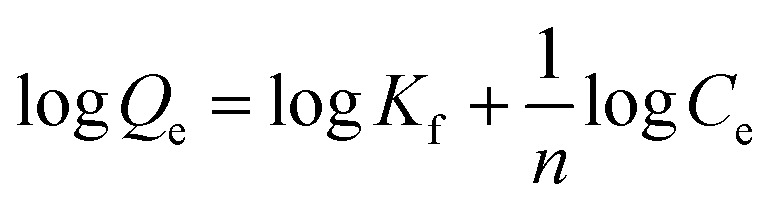
*K*_f_ is the Freundlich constant, while *n* gives the value of the degree of linearity between the adsorbate and the adsorption process.

The following equation is employed to represent the Temkin isotherm:^27^

9 *Q*_e_ = *B*_T_ ln *A*_T_ + *B*_T_ ln *C*_e_

where *B*_T_ is the coefficient of adsorption heat and *A*_T_ is the bonding equilibrium constant.

### 3.2 Kinetics study

The kinetic behavior of synthesized materials for the adsorption of moisture was assessed using both “Lagergren's” first-order adsorption kinetics model and second-order adsorption kinetics model, respectively. Both models were utilized by employing the below parameters. The water content at time *t* and its equilibrium moisture content can be calculated:10
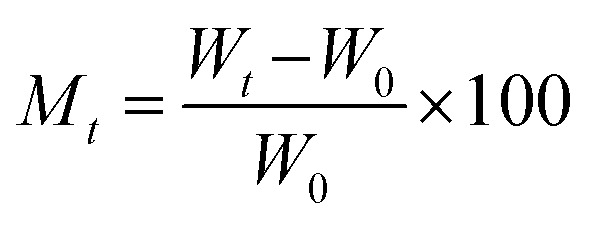
11
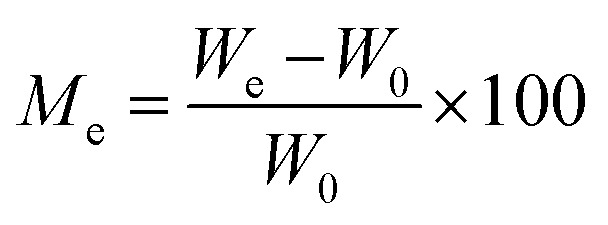
where *W*_0_ is the initial weight of a sample, *W*_*t*_ is the weight of the sample at the adsorption time *t*, *W*_e_ is the weight of the sample at the equilibrium.

The first-order adsorption kinetics model was developed to elucidate the behavior of adsorption. The adsorption rate was believed to be dependent on the amount of adsorption, with the variation in adsorption amounts acting as the primary driving force for adsorption. The first-order kinetic model is represented by the corresponding equation:

12 *M*_*t*_ = *M*_e_ + (*M*_O_ − *M*_e_)e^−*k*_1_*t*^

The parameter *k*_1_ represents the adsorption rate constant for first-order kinetics, measured in units of h^−1^.

The second-order kinetics model was employed to elucidate the process of adsorption, which comprises chemical reactions involving the formation and breaking of molecular bonds. The calculation of the equilibrium adsorption quantity, *M*_e_, may be determined using the following equation:13
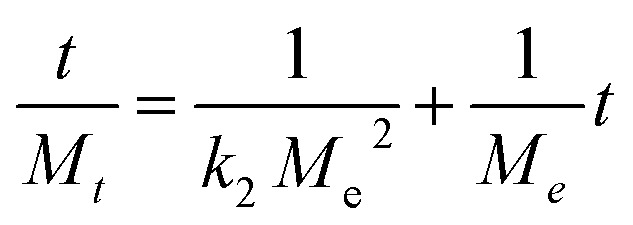
*M*_*t*_ is the adsorption capability at any time *t*, *M*_e_ denotes the adsorption at equilibrium, and *M*_o_ is the adsorption at *t* = 0. *k*_2_ is the adsorption rate constant for a second-order kinetic model.

Both models were applied using the following parameters. The pseudo-second-order kinetic model was well fitted to the as-synthesized materials as compared to the first-order kinetic model.

### 3.3 Thermodynamic studies

The thermodynamic study is based on several parameters. These parameters are isosteric heat of adsorption, Gibbs free energy, and entropy.

14 *Q*_st_ = *q*_st_ + *H*_L_

15
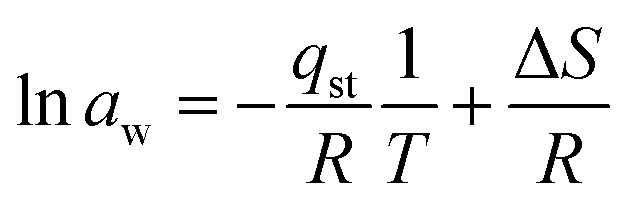

16 Δ*G* = *RT* ln *a*_w_

They provide information about the adsorption process, whether it is exothermic, endothermic, non-spontaneous, or favorable. The negative value of Δ*G* reveals that the process of adsorption is spontaneous, while the positive value shows that the process is endothermic.^28^

## 4. Results and discussion

### 4.1 Characterization

**Fig. 2:**
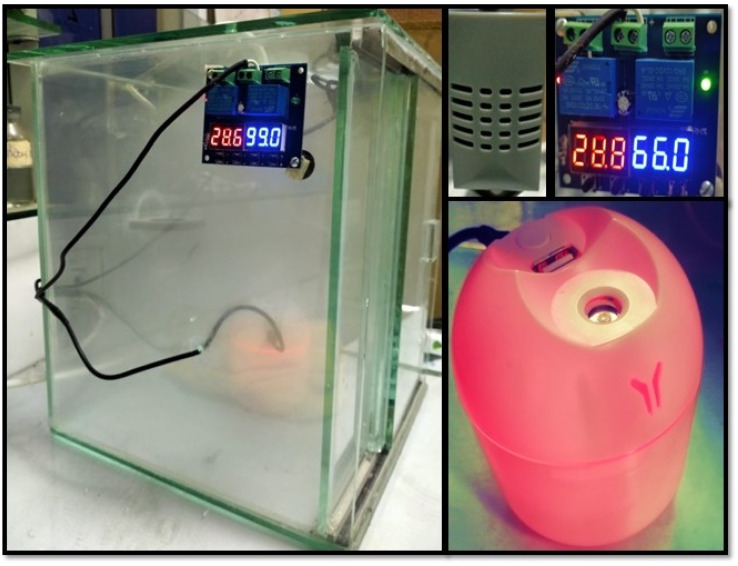
Indigenous atmospheric water harvesting plant.

The synthesized samples of MOF-5, GO, and the MOF-5/GO composite were characterized by XRD analysis to study phases. The XRD pattern of synthesized materials is shown in Fig. 2. The sharp and intense peaks of GO are observed with *hkl* values of (001,002,100), respectively. At 26°, typical (002) planes of graphite are seen (ICSD File. 75-2078). The diffraction patterns of the graphene oxide samples show a peak at around 11°, corresponding to the (001) plane, and another at about 43° with the (100) plane (JCPDS No. 82-2261). In addition, the GO interfacial distance was raised with a *d*-spacing of 0.77 nm (FWHM = 0.4368). This increase was caused by the incorporation of oxide functional groups, such as OH, C–O, C=O, and COOH, during chemical oxidization reactions at the graphite basal planes. As a result, there was a large gap between the succeeding carbon layers.^29^

**Fig. 3:**
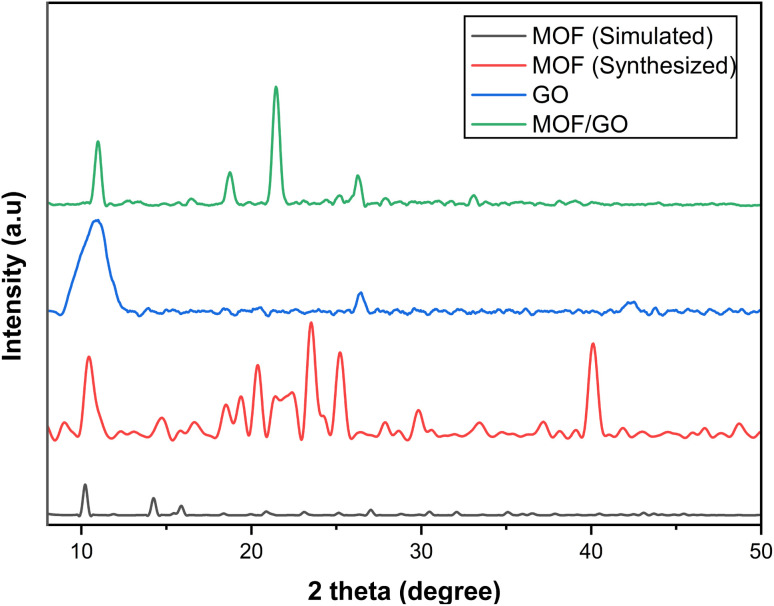
X-ray diffraction (XRD) patterns for the parent and composite materials.

The diffraction patterns of MOF-5 are in agreement with the previously reported pattern by Yaghi *et al.*^30^ and that reported by Huang *et al.*^31^ The observed patterns are also closely related to the single crystal pattern (CCDI File No. 1516287). MOF-5 shows sharp peaks at 2 theta values of 10.72°, 21.3°, 23.9°, 25.53°, and 41.57°, with *hkl* values of 220, 660, 640, 731, and 882, respectively. Several patterns are also observed between 30° and 40°, which might be the phase of free zinc oxide.^32^ There is somehow a change in the peak position and intensity, which is due to the different crystal growth and atomic orientation, but they do not change the crystal structure of the material. A diffraction peak at 2*θ* of 10°, 18°, 21°, 25°, and 32° is seen in GO/MOF-5 (Fig. 3). Since MOF-5 is our primary component in the hybrid, the GO/MOF-5 diffraction pattern resembles that of MOF-5, suggesting that the MOF-5 structure is retained and does not undergo GO-induced deformation.

The prepared samples were characterized by FTIR to determine the functional groups and chemical structure. The FTIR spectra of GO, MOF-5, and GO/MOF-5 are similar to those reported in the literature.^33^ For graphene oxide, the existence of an O–H group was confirmed by the medium-sharp peak that emerged at a wavelength of 3650 cm^−1^. Additionally, at a frequency of 1612 and 1714 cm^−1^, the C=C and C=O stretching was observed, while the CO stretching was observed at 1016 cm^−1^. Since COOH, OH, C=O, and C–O groups were present, it was determined that oxygen atoms were heavily occupied along the edges and basal plane of GO, indicating the confirmation of graphene oxide.^29^

**Fig. 4:**
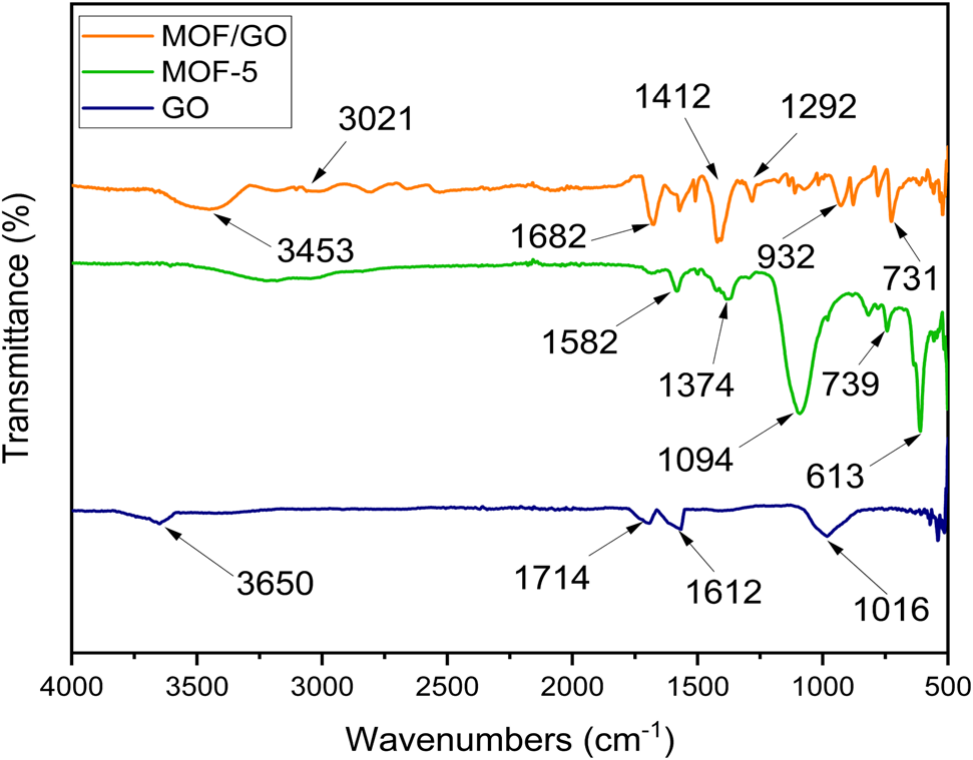
FTIR spectra of GO, MOF-5, and the GO/MOF-5 composite.

In the FTIR spectrum of MOF-5, the peaks before 1000 cm^−1^ show the presence of metal oxide. The peak appearing around 1370 cm^−1^ is caused by symmetric stretching of the carboxylic group in BDC, while the peak appearing at 1582 cm^−1^ shows the asymmetric stretching of the carboxylic group. In GO/MOF-5, the peaks at 1292 cm^−1^, 1412 cm^−1^, and 1682 cm^−1^ represent C–O, OH bending, and C=O, respectively, whereas the band at 3021 cm^−1^ corresponds to C–H bending. The broad band at 3453 cm^−1^ must be due to the overlapping bands from OH, indicating the presence of water content on a sample (Fig. 4).^34^

The surface morphology of the synthesized composite was revealed by scanning electron microscopy. It is observed from Fig. 2(a and b) that the GO and MOF-5 sample surfaces contain spherical particles of different sizes.

**Fig. 5:**
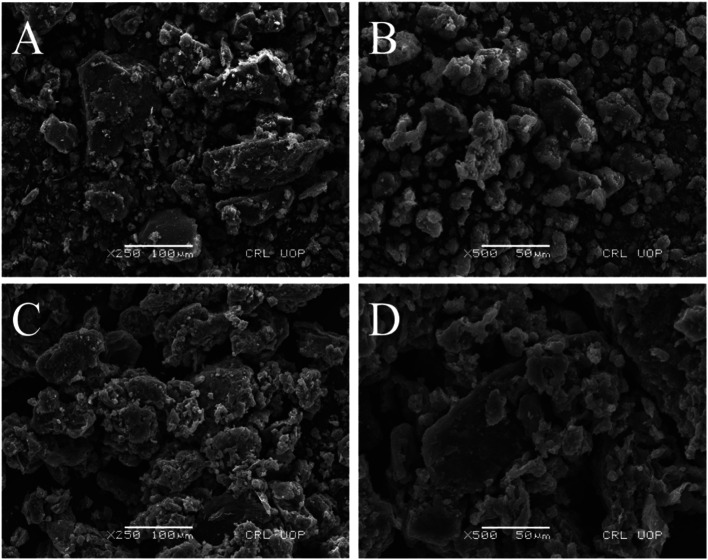
SEM image of (A) GO, (B) MOF-5, and (C and D) GO/MOF-5.

From Fig. 5(c and d), it is clearly seen that the incorporation of GO into MOF-5 increases the overall porosity of the GO/MOF-5 composite. The high-water vapor adsorption capacity of the GO/MOF-5 composite might be due to the increase in functional group and porosity.

Points of zero charge (PZC) were employed to analyze the charge on the surface of the materials. The exterior charge of materials is important for studying the mechanism of water interaction with materials. The salt addition method was applied to ascertain the point of zero charge. The determination of the point of zero charges (pH_pzc_) of prepared samples is presented in Fig. S1.† The pH_pzc_ of graphene oxide is 6.9, that of MOF-5 is 6.0, and that of GO/MOF-5 is 1.83, which is less than the pH of water (7.4), as pH > (pH_pzc_), which indicates that the charge on the surface of all materials is negative. The negative charge on the surface of materials might be due to the presence of various functional groups such as hydroxyl, epoxy, ester, and carboxylic groups.

### 4.2 Moisture adsorption studies

The moisture content of all the samples was determined using the same process as described in Section 2.6. The plots of moisture content (Mc) and time were plotted using Origin software and MS Excel. Initially, the sites on the material surface are accessible for adsorption; with time, sites are occupied by water molecules, and equilibrium is achieved. The point at which equilibrium is established is known as the saturation point. The state in which material neither absorbs nor desorbs moisture is called equilibrium moisture content (EMC). After the sorption point, the adsorption of the material starts to decrease. The adsorption of water vapor depends on the RH%. At different RH%, different moisture contents of materials are obtained.

When considering graphene oxide (GO), the moisture content rises as the relative humidity (RH%) rises. This is happening since there are many sites available for binding owing to the many functional groups that readily capture water molecules. On the other hand, the Mc value in the case of MOF-5 increases little at first. Unexpected fluctuations in the water content of MOF-5 are noticed at a relative humidity of 55%. This is followed by a further decrease in water content at a relative humidity of 61%. The rapid rise in moisture content of MOF-5 between RH 43% and RH 63% may be attributed to its unstable nature toward higher humidity levels. This is caused by an abrupt breakdown in their structure caused by weak metal oxide bonds, which ultimately triggers a hydrolysis process in MOF-5.^35^ When compared to MOF-5, the GO/MOF-5 composite shows a significant rise in moisture content; at 75% relative humidity, the maximum moisture value of 1137 mg g^−1^ is achieved.

**Fig. 6:**
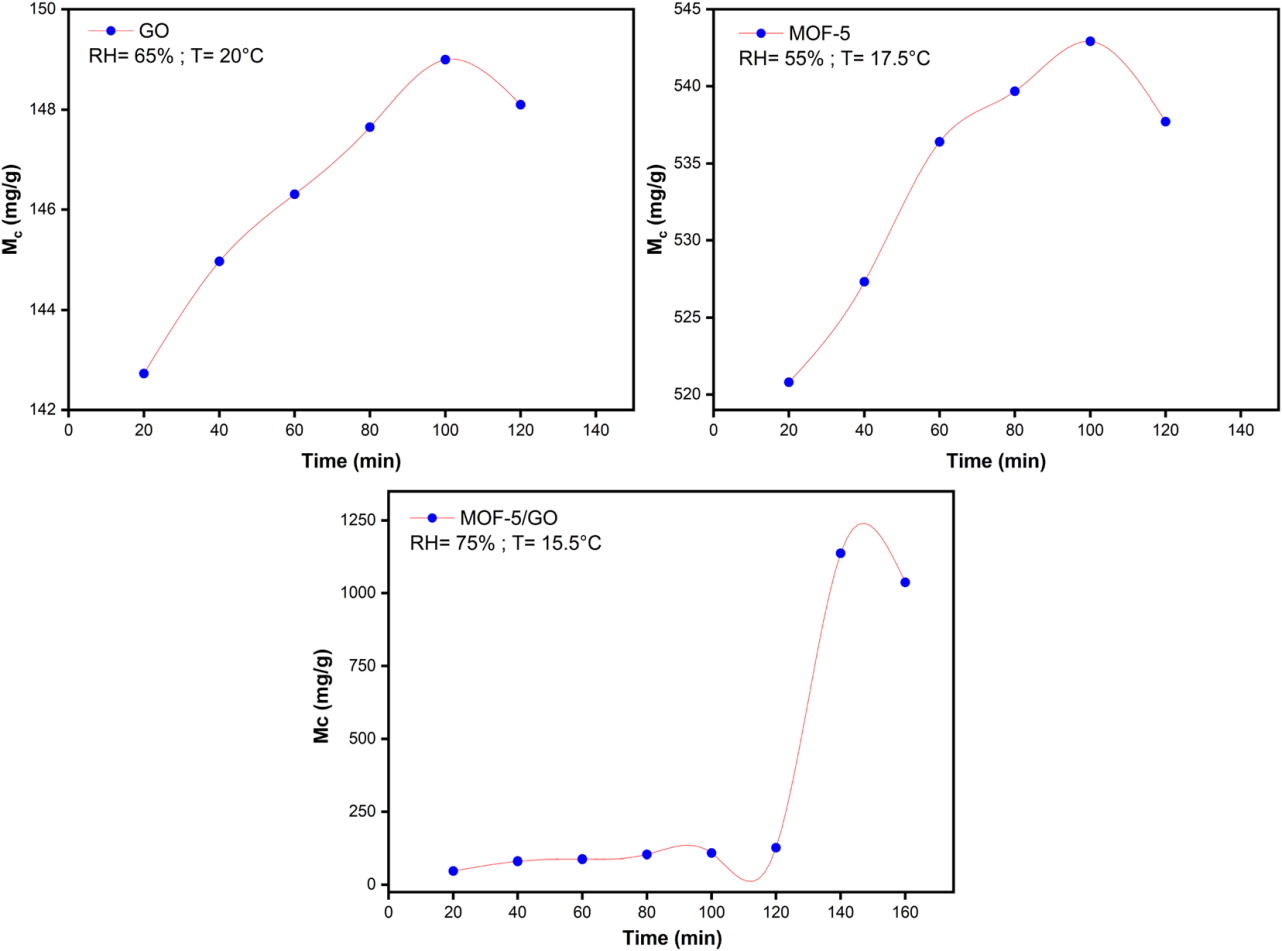
Maximum moisture content (Mc) study against time (min) on GO, MOF-5, and the GO/MOF-5 composite.

At 75% RH, exceptional behavior in water uptake performance for the GO/MOF-5 composite is observed. This is due to the capillary condensation process that takes place at the surface of the material. From 0 to 120 min, the water molecule binds to the functional group at primary adsorption sites through hydrogen bonding. From 120 to 140 min, a steep rise is observed in the case of the GO/MOF-5 composite. This reveals that there is a pore filling of material that takes place at this stage. Beyond 140, all the available sites are filled with water vapor, so there is no further increase in moisture content (Fig. 6).^36–38^

Based on the studies regarding moisture content, GO is making MOF-5 more hydrolytically stable and increasing the adsorption capability of the GO/MOF-5 composite. Due to the strong pi–pi interactions and decreased repellent hydration forces between the sheets of GO, GO exhibits strong hydrophilicity and quick water dynamics.^39,40^ These properties lead the MOF-5 to become more durable in water.

#### 4.2.1 Structural stability of GO/MOF-5 composite

**Fig. 7:**
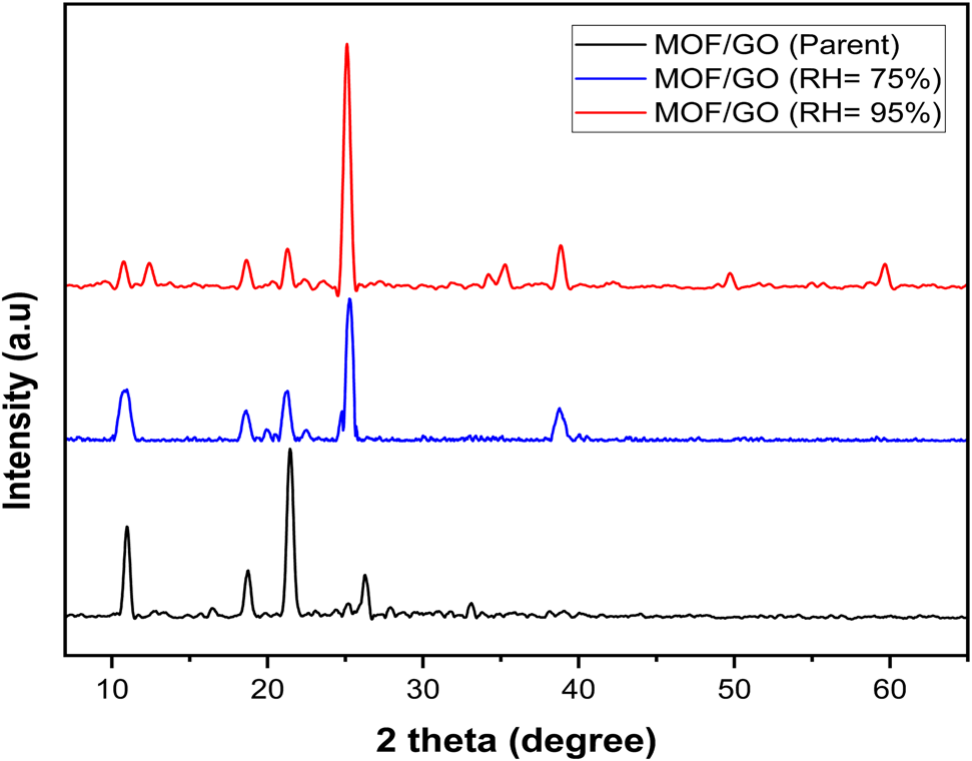
X-ray diffraction (XRD) patterns for the GO/MOF-5, GO/MOF-5 (75%) and GO/MOF-5 (95%).

The structural stability of GO/MOF-5 composite was evaluated by XRD analysis at the RH of 75% and 95%. It is observed from Fig. 7 that the XRD pattern of GO/MOF-5 composite is closely matched with the GO/MOF-5 composite (75% and 95%). This means that there are no structural changes taking place even at higher humidity of 75% and 95%.^41^

#### 4.2.2 Water adsorption mechanism in MOF-5

There are twenty distinct adsorption sites on metal clusters and twelve on linkers, discovered by Ming and coworkers. Considering that each of the water molecules primarily fills the most actively favorable adsorption sites on the Zn–O cluster.^42^ Three modes of interaction between water molecules and MOF-5 are illustrated by Greathouse: (1) by directly replacing one of the coordinating MOF O atoms (O1 or O2) with the oxygen of the water molecule; (2) hydrogen bonding the hydrogen atom of the water molecule with O_2_; and (3) by creating a network of the water molecules that are tethered (hydrogen-bonded) to one or more ZnO_4_ tetrahedra.^43^ Briefly, water molecules adsorb in MOF-5 into the tight pores of ZnO or form bonds with ZnO. Water molecules bonded to zinc through the Zn–O interaction between metal clusters and organic linker being broken, forming an OH group. The leftover hydrogen then forms a bond with the linker to produce a carboxylic acid.

### 4.3 Adsorption isotherms

The adsorption isotherm constitutes one of the crucial elements of adsorption. The phenomenon and interactions among adsorbate and adsorbent are explained by the adsorption isotherms. The adsorption isotherm model may offer information on the adsorbent's adsorption capacity, the mechanism of adsorption, and the assessment of the adsorption process's performance. The obtained data were fitted to various adsorption isotherms to determine the nature of the adsorption.

**Fig. 8:**
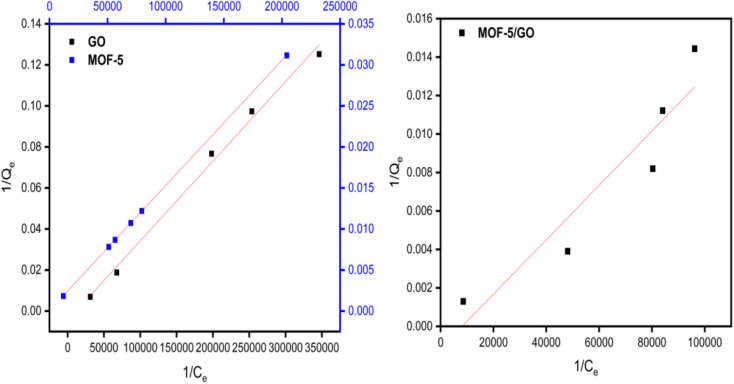
Study of the Langmuir model on GO, MOF-5, and the GO/MOF-5 composite.

According to the Langmuir isotherm, the presence of a single layer of adsorbate on the adsorbent surface causes the maximum adsorbent capacity to occur. The following equation represents the isotherm of Langmuir.^27^ A value of *R*_L_ >1 is not a favorable adsorption mechanism, *R*_L_ = 1 is straight, while *R*_L_ = 0 is an irreversible mechanism of adsorption (Fig. 8).

**Fig. 9:**
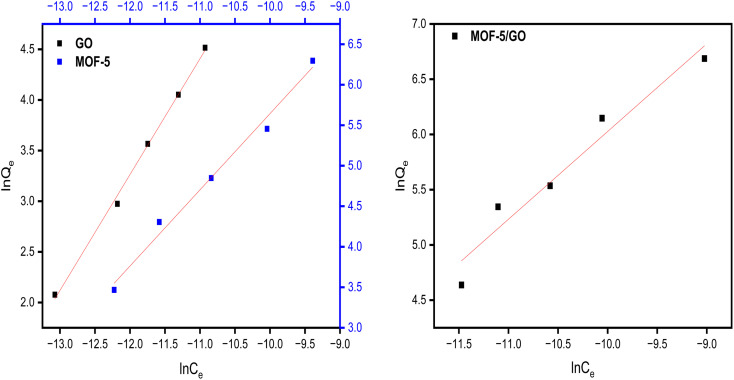
Study of the Freundlich model on GO, MOF-5, and the MOF-5/GO composite.

Freundlich isotherms describe a physical adsorption known as multilayer adsorption, where the interactions between the molecules are weak and the adsorption takes place in many layers. The value of *n* describes linear adsorption when *n* = 1, physisorption when *n* > 1, and chemisorption when *n* < 1. The Freundlich model fits well with all materials (Fig. 9).

**Fig. 10:**
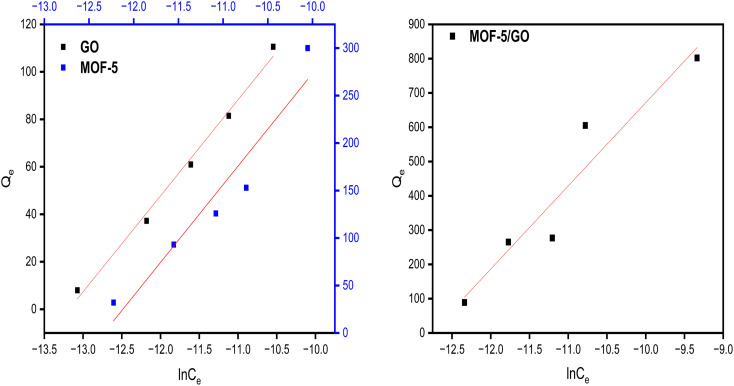
Study of the Temkin model on GO, MOF-5, and the GO/MOF-5 composite.

Temkin model for all samples presented in (Fig. 10). The Temkin isotherm presupposes three assumptions: the impact of indirect adsorbate/adsorbate correlations on the adsorption procedure; homogenous spreading of binding energy; and adsorption heat of atoms falling linearly with the coverage of the adsorbent surface.

*B*_T_ is the coefficient of adsorption heat, and *A*_T_ is the bonding equilibrium constant. However, the *B*_T_ value provides details on the chemisorption or physisorption process. The process of adsorption occurs physically if *B*_T_ < 8 kJ mol^−1^ and chemically when *B*_T_ > 8 kJ mol^−1^.

The parameters of all isotherm model were compared, along with the *R*-square values (Table 1). It was concluded that there is linear sorption and chemisorption behavior in every material based on the isotherm study.

**Table 1:** Parameters of isotherm models for GO, MOF-5, and the GO/MOF-5 composite

Material	Langmuir	Freundlich	Temkin
	*R* ^2^/*R*_L_	*R* ^2^/*n*	*R* ^2^/*B*_T_ (kJ mol^−1^)
GO	0.994/0.52	0.997/1	0.99/40.43
MOF-5	0.99/0.99	0.99/1	0.92/11.70
GO/MOF-5	0.887/1.00	0.88/1	0.91/24.26

### 4.4 Kinetics study

**Fig. 11:**
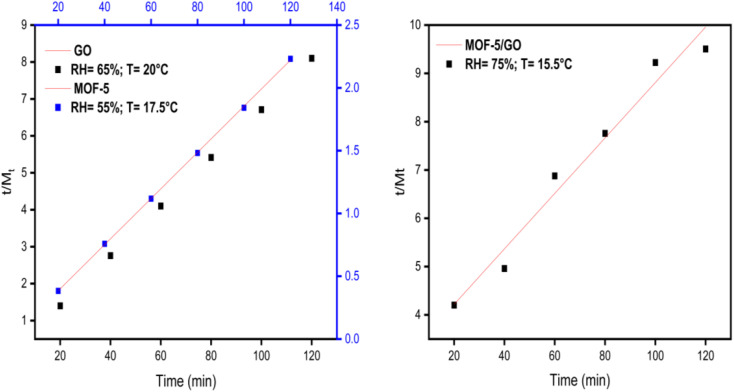
Pseudo-second-order kinetics study on GO, MOF-5, and GO/MOF-5.

Plotting *t*/*M*_*t*_*vs.* time (*t*) results in a straight line with values for the intercept, slope, *M*_e_, *R*^2^, and second-order rate constant (Fig. 11). The greater value of the linear regression correlation coefficient *R*^2^ was obtained using the second-order kinetics model. As a result, the experimental results and parameters have a very strong correlation. Second-order kinetics also reveal that the material exhibits chemisorption behavior. The values of the slope, intercept, second-order rate constant (*k*_2_), and experimental and theoretical *M*_e_ for GO, MOF-5, and the GO/MOF-5 composite are given in Tables S2–S4 and 2.†

**Table 2:** Parameters of pseudo-second-order kinetics for GO, MOF-5, and GO/MOF-5

Material	RH%	Slope (*m*)	Intercept (*c*)	*M*_e_ (Th)	*M*_e_ (Exp) (mg g^−1^)	*k*_2_ (min^−1^)
GO	65 ± 3	0.0666	0.0808	14.8993	14.9927	0.0550
MOF-5	55 ± 3	0.01836	0.0176	54.2913	54.4662	0.1915
GO/MOF-5	75 ± 3	0.05742	3.06936	13.7475	17.4155	0.01074

### 4.5 Thermodynamics study

#### 4.5.1 Isosteric enthalpy of adsorption

Isosteric enthalpy of adsorption measures the difference in heat of adsorption at a specific adsorbate condition and temperature. The heat of vaporization for water is (*H*_L_, 43 kJ mol^−1^). The value of *Q*_st_ is found using the following equation:

17 *Q*_st_ = *q*_st_ + *H*_L_

The Clausius–Clapeyron equation was used to drive it by the graph of 1/*T vs.* ln *a*_w_.18
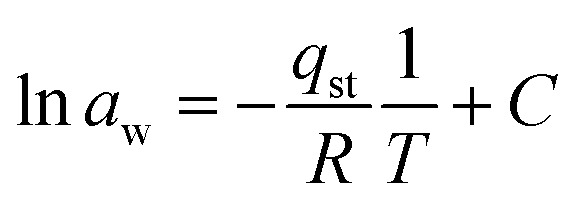
In this equation, *q*_st_ is the absolute isosteric heat of sorption in joules per mol, *T* expresses the absolute temperature, *R* expresses the molar gas constant, and *C* is the constant.

Using the slope derived from the equation, the isosteric heat of adsorption (*Q*_st_) was calculated.19
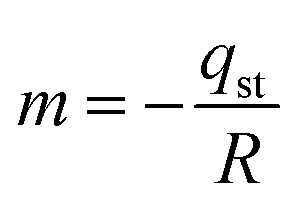


#### 4.5.2 Sorption entropy

Entropy change provides details about the measurement of energy since it is inversely related to the number of adsorption sites that are accessible. The equation may be used to compute Δ*S* by plotting ln(*a*_w_) and 1/*T* and taking the intercept as Δ*S*/*R*.20
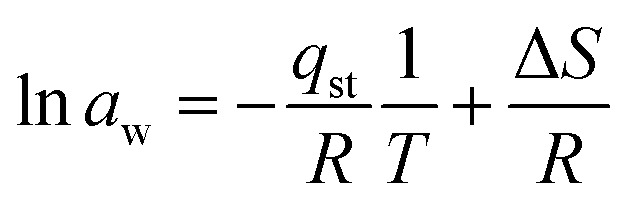
(*C* = −27.20; *R* = 8.314 J mol^−1^ K^−1^)21
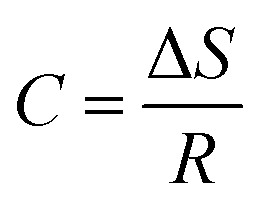
Δ*S* = −27.20 × 8.314Δ*S* = −0.2261 kJ mol^−1^

#### 4.5.3 Gibbs free energy

The amount of energy released during a process with constant pressure and temperature is given by the Gibbs free energy. It is possible to determine whether a process is spontaneous or non-spontaneous by the value of Δ*G*. The following equation is used for the calculation of Δ*G*.

22 Δ*G* = *RT* ln *a*_w_

23 Δ*G* = *RT* ln *a*_w_

Δ*G* = 8.314 × 289.4 × ln(0.75)Δ*G* = −6.91 kJ mol^−1^

The value of Δ*G* for the GO/MOF-5 composite is negative, so the process of adsorption is spontaneous in nature.

The change in Gibbs free energy (Δ*G*) with Xeq for GO, MOF-5, and the GO/MOF-5 composite is represented in Fig. S8–S10.† The Δ*G* for synthesized material varied from −1.95 to −1.22 kJ mol^−1^. The negative value for all samples confirms that the water vapor adsorption process is spontaneous at different humidity levels.^28^

## 5. Conclusion

Over the past few years, MOFs and MOF-based composites have received great attention in wastewater treatment and AWH. MOFs and MOF-based composites act as effective adsorbent materials due to their high porosity, tunable pore sizes, easy functionalization, high adsorption capacity, and surface area, making them ideal for the adsorption process. In the present study, we synthesized GO, MOF-5, and a GO/MOF-5 composite and evaluated their behavior toward water vapor adsorption. The synthesized materials were characterized by XRD, SEM, and FTIR analysis. XRD analysis confirms the successful synthesis of the materials. SEM analysis confirms the formation of well-shaped particles and a porous GO/MOF-5 composite. In FTIR analysis, the peak observed at 3000–3650 cm^−1^ confirms the presence of water vapor adsorption on all the synthesized samples. PZC confirms the presence of a negative charge on all synthesized samples. MOFs are stable at lower humidity values and show structure changes at higher humidity values. MOF-5 shows a Mc value of 29 mg g^−1^ and 542 mg g^−1^ at a RH of 45% to 95%. At 65% RH, the Mc value of MOF-5s greatly decreased from 542 mg g^−1^ to 81 mg g^−1^. The incorporation of GO imparts great structural strength to MOF-5 at higher humidity values. The Mc values of the GO/MOF-5 composite increased from 89 mg g^−1^ to 1137 mg g^−1^ at 45% to 95% RH. The results evaluated from the isosteric heat of adsorption, entropy, and Gibbs free energy suggest that the water vapor adsorption process was exothermic and spontaneous. The second-order kinetics model confirms the chemisorption behavior of all the synthesized materials. The high adsorption capacity of the GO/MOF-5 composite makes it ideal for applications in AWH.

## Author contributions

Muhammad Saeed-Ul-Hassan: conceived and designed the experiments, performed the experiments, analyzed the data, prepared figures and/or tables, and wrote the original draft. Muhammad Ehtisham: conceived and designed the experiments, analyzed the data, and wrote the original draft. Bushra Ismail (CA): Supervised, provided chemicals, conceived and designed the experiments, analyzed the data, authored or reviewed drafts of the article, and validated and approved the final draft. Ahmad K. Badawi: authored or reviewed drafts of the article, edited, and validated and approved the final draft. Asad Muhammad Khan: provided chemicals and performed XRD and analyzed the results. Rafaqat Ali Khan: performed and analyzed the FTIR analysis.

## Conflicts of interest

There are no conflicts to declare.

## Supplementary Material

NA-006-D4NA00150H-s001
