# Supplementary material for: A comparative study of moisture adsorption on GO, MOF-5, and GO/MOF-5 composite for applications in atmospheric water harvesting
Source: Nanoscale Adv. 2024 Jun 7;6(14):3668–79. doi: 10.1039/d4na00150h (PMC11232537; doi:10.1039/d4na00150h)
Supplement: NA-006-D4NA00150H-s001 [file NA-006-D4NA00150H-s001.pdf]

Electronic Supplementary Material (ESI) for Nanoscale Advances.
This journal is © The Royal Society of Chemistry 2024

Point of Zero charge

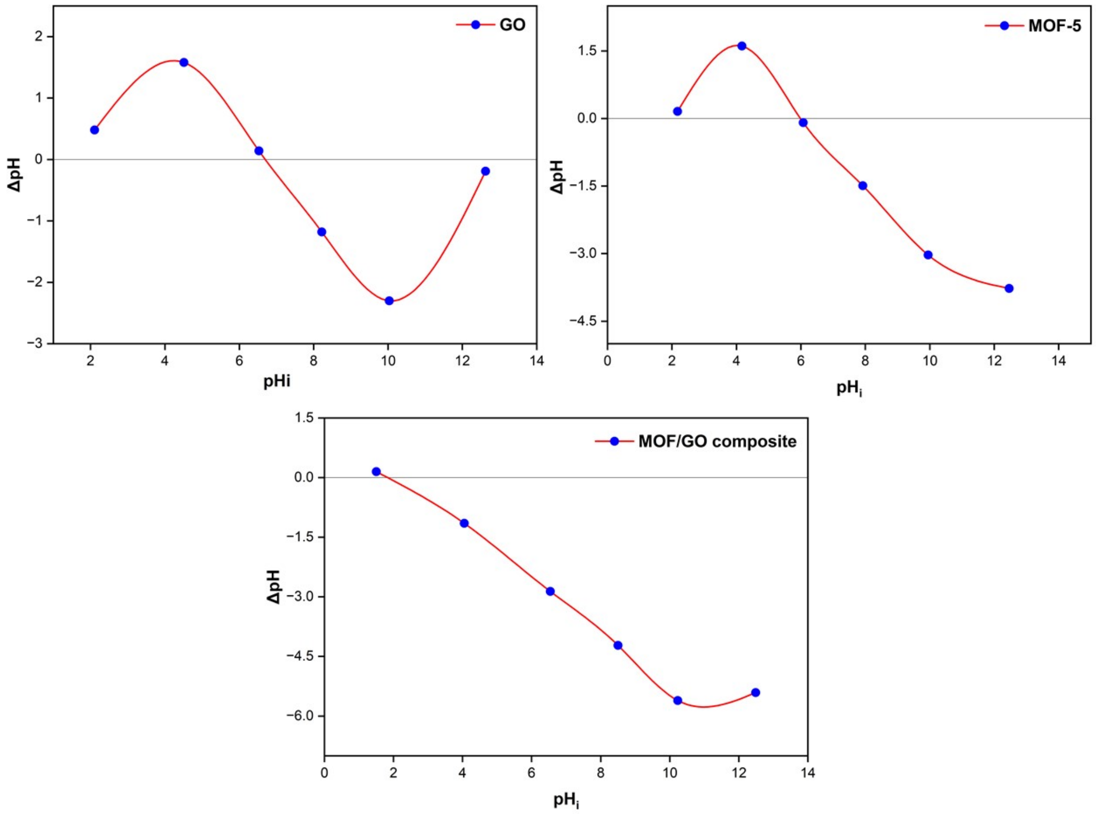

Figure S1: Change in pH versus initial pH for GO, MOF-5, and GO/MOF-5

Moisture Content Graphs

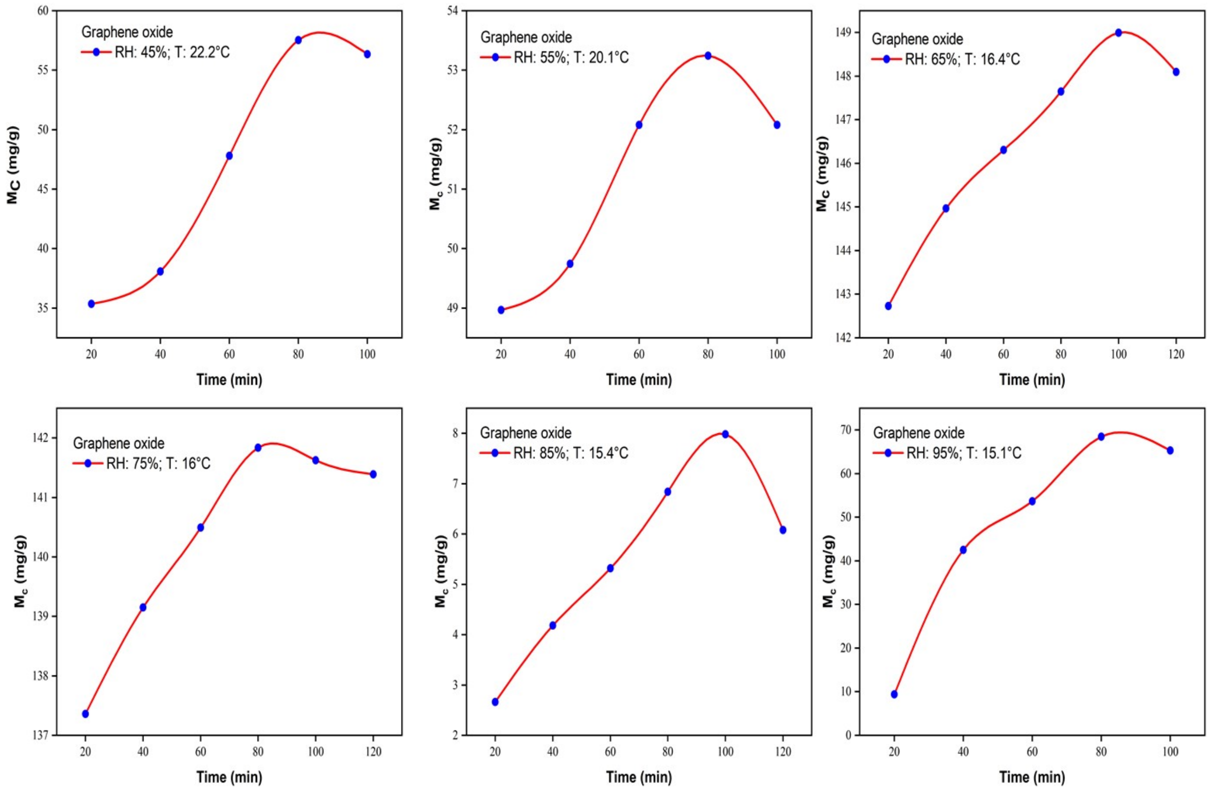

Figure S2: Moisture Content (Mc) against time for synthesized GO at different RH%

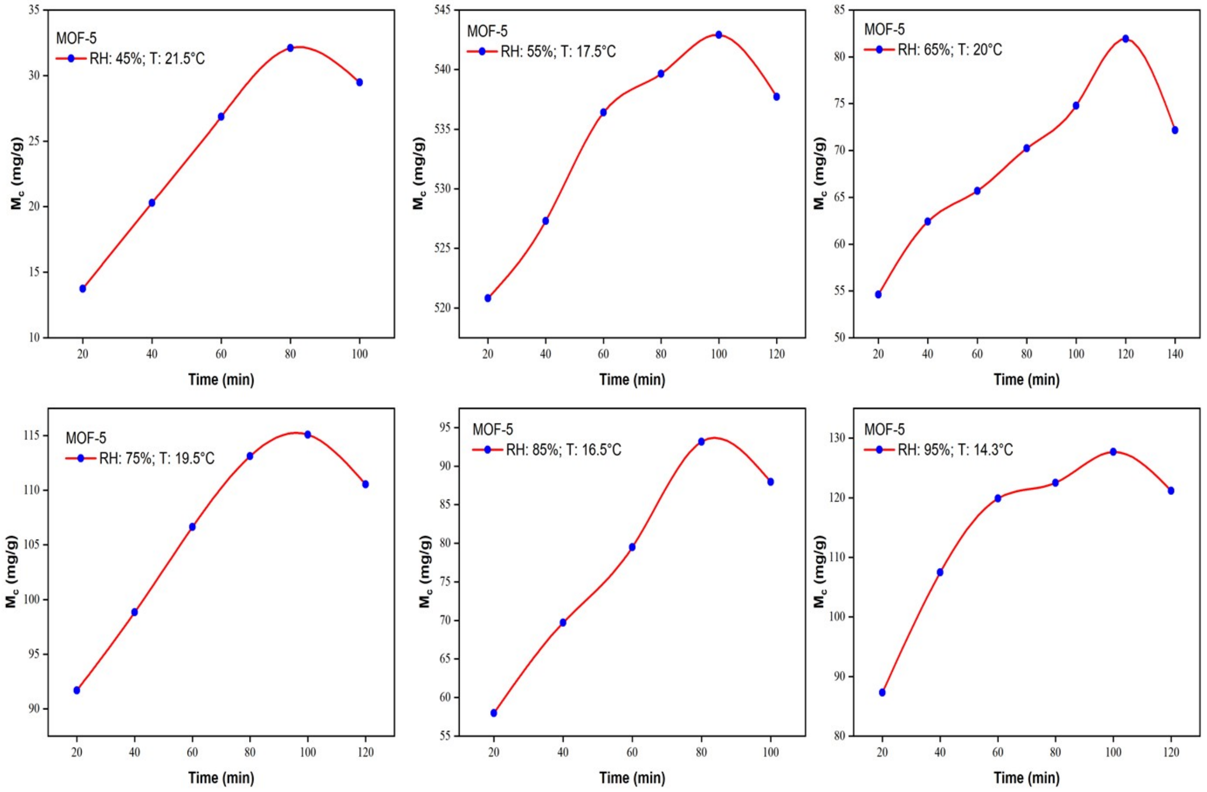

Figure S3: Moisture Content (Mc) against time for synthesized MOF-5 at different RH%

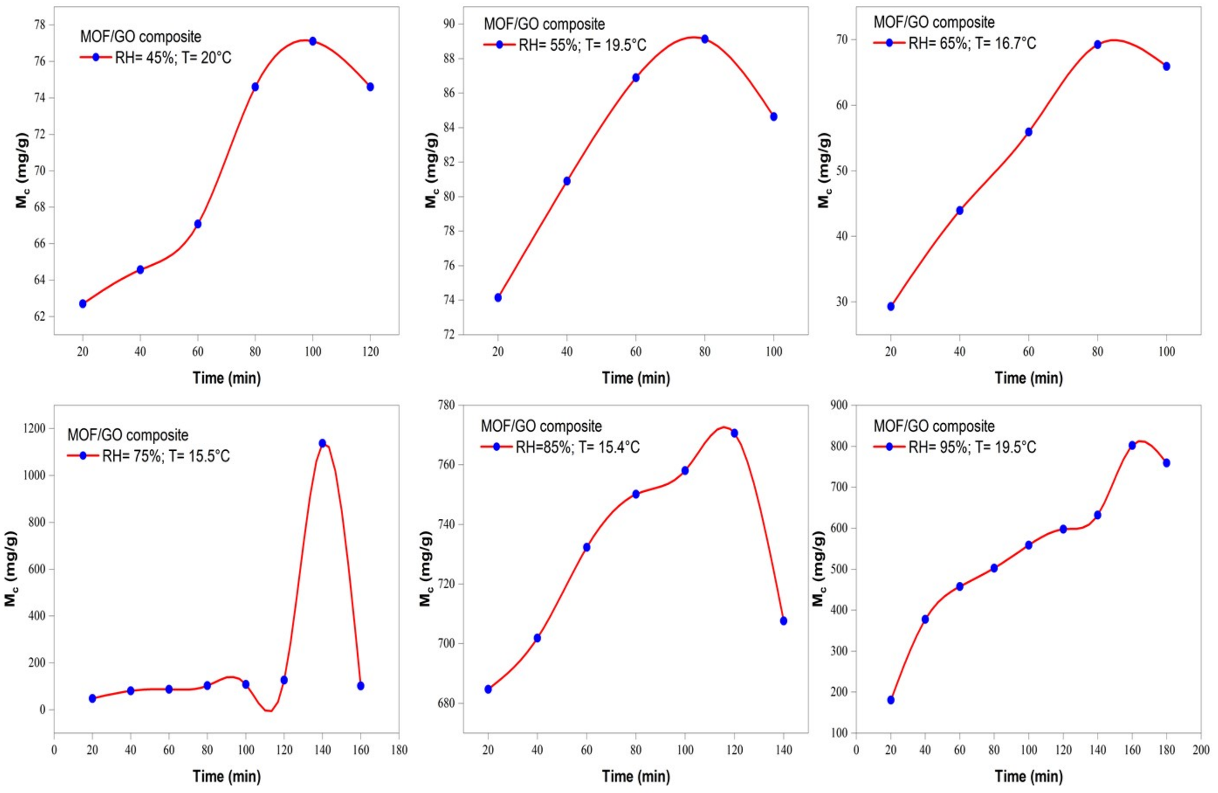

Figure S4: Moisture Content (Mc) against time for synthesized GO/MOF-5 at different RH%

Equilibrium Moisture Content (Xe) v/s Water Activity

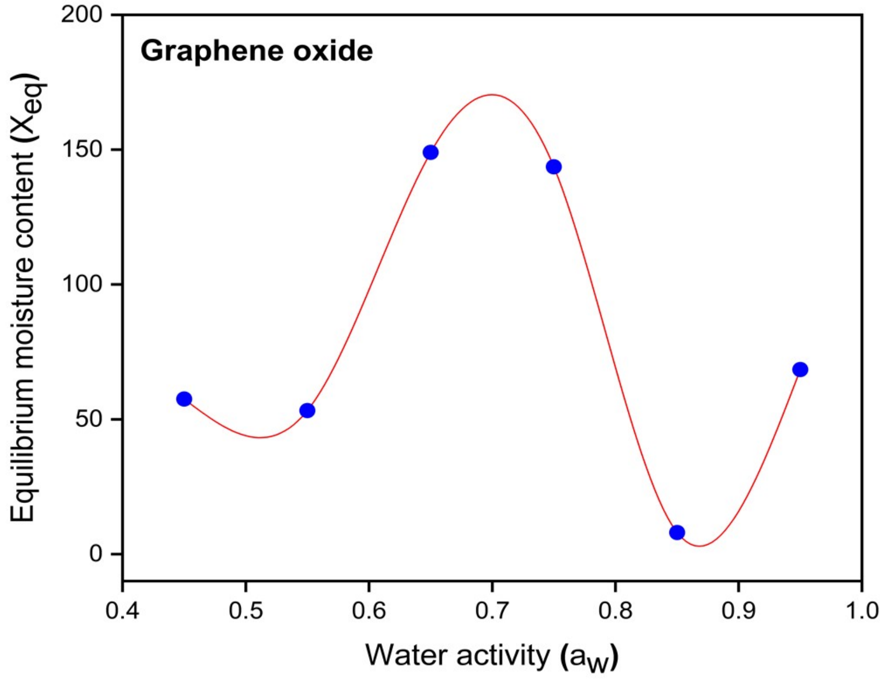

Figure S5: Xeq versus aw for synthesized graphene oxide

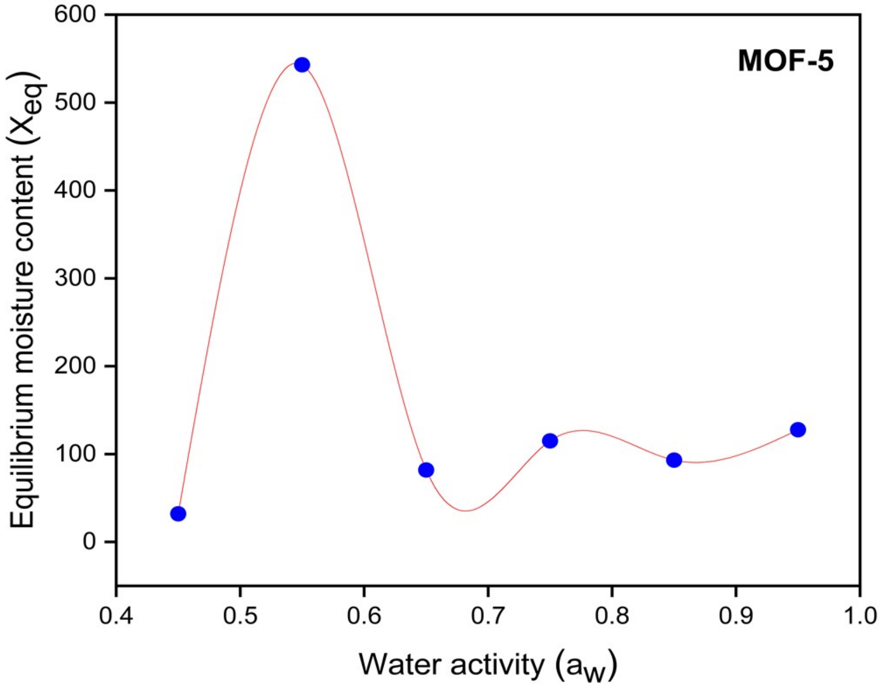

Figure S6: Xeq versus aw for MOF-5

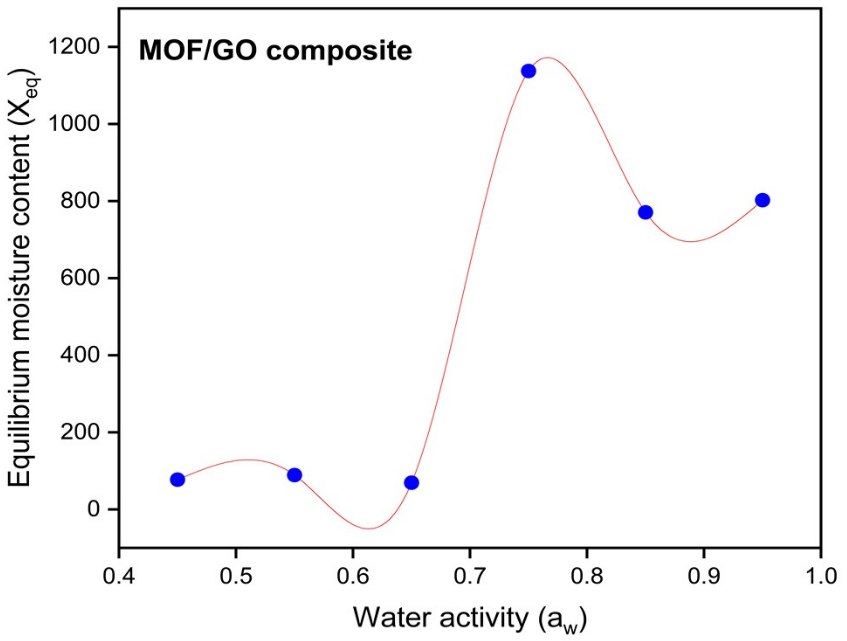

Figure S7: Xeq versus aw for GO/MOF-5

Thermodynamics study of GO:

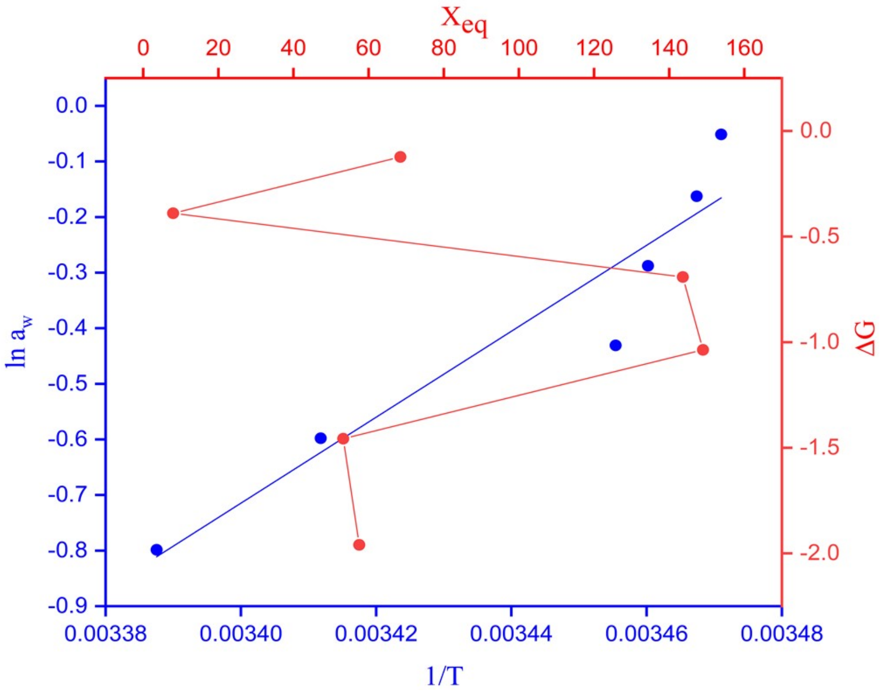

Figure S8: Relationship between change in Gibbs free energy versus X_{eq} and ln a_w versus 1/T for GO

Thermodynamics study of MOF-5:

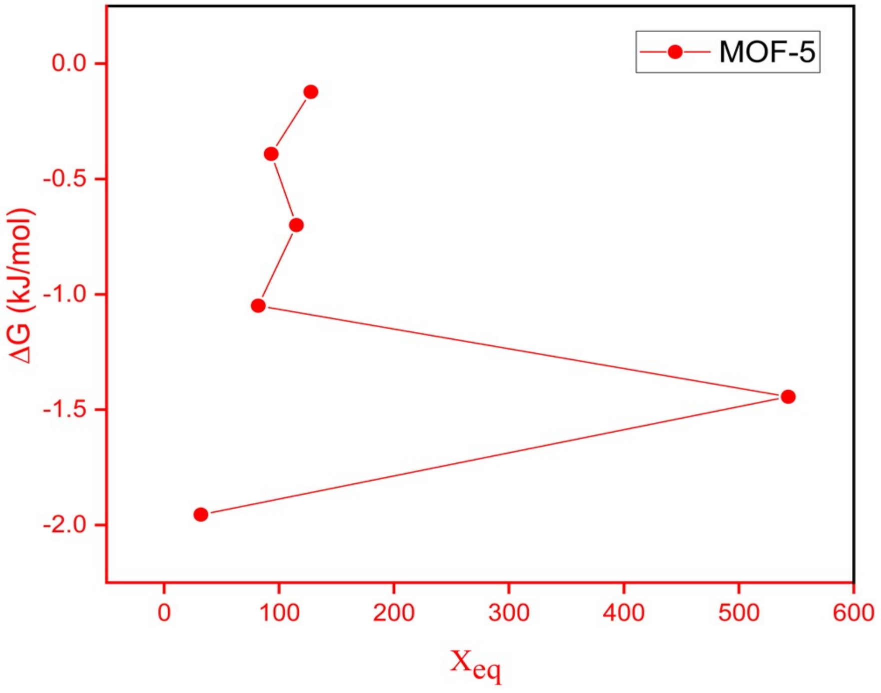

Figure S9: Relationship between change in Gibbs free energy versus X_{eq} for MOF-5

Thermodynamics study of GO/MOF-5 Composite:

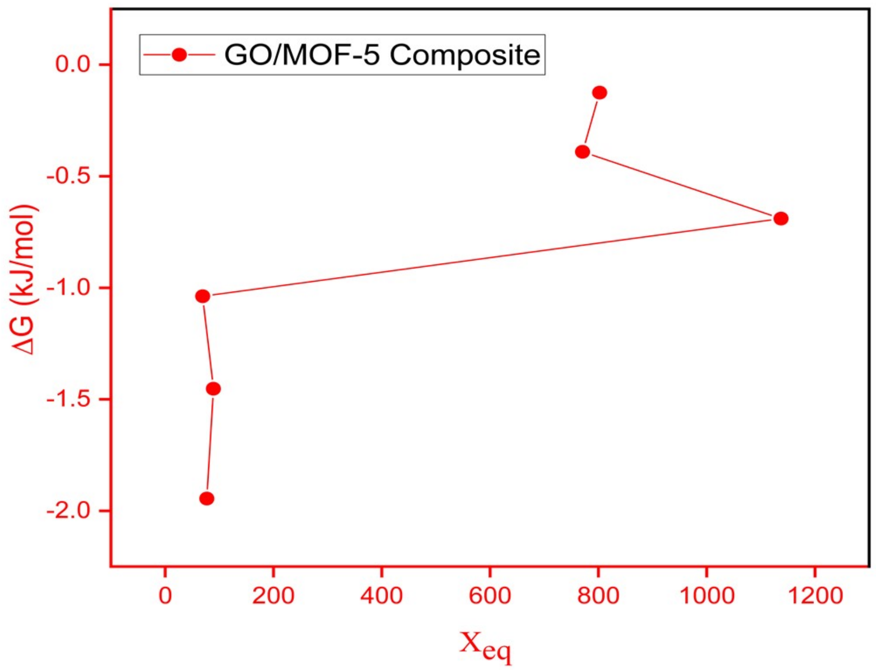

Figure S10: Relationship between change in Gibbs free energy versus X_{eq} for GO/MOF-5 composite

Pseudo 2nd order kinetic Graphs

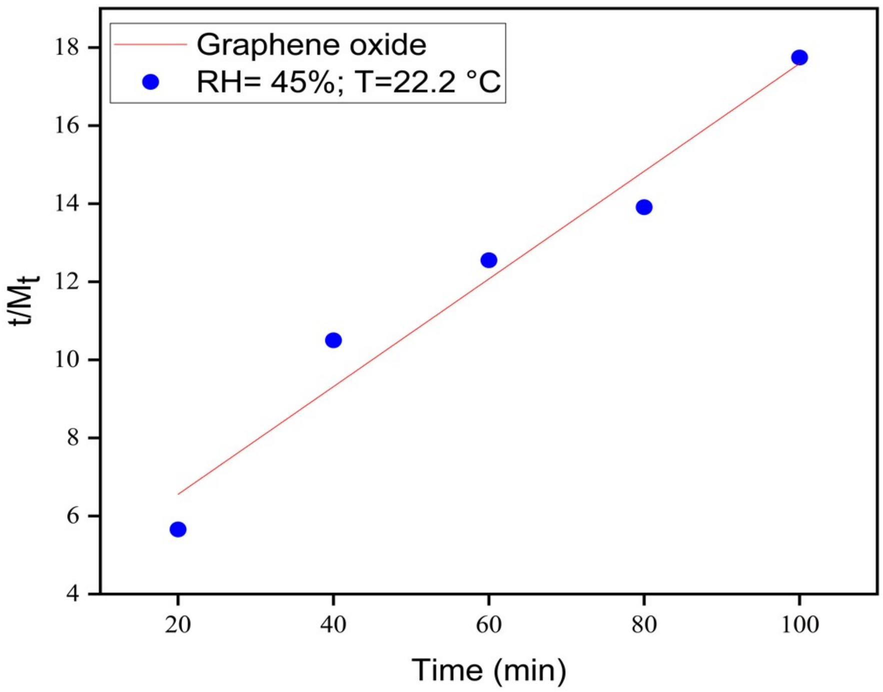

Figure S11: Pseudo 2nd order kinetics model for synthesized GO at a RH of 45 %

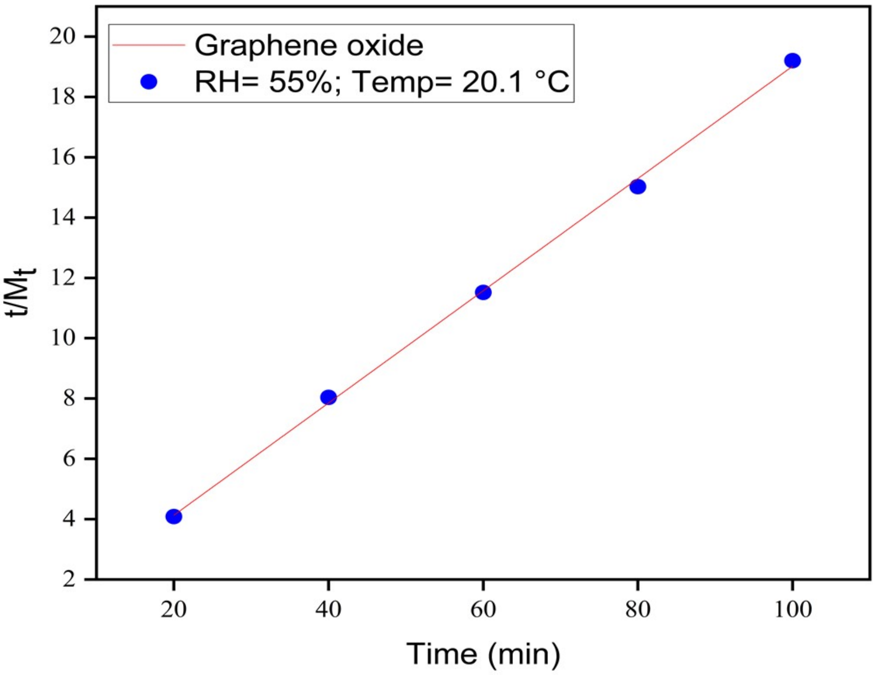

Figure S12: Pseudo 2nd order kinetics model for synthesized GO at a RH of 55%

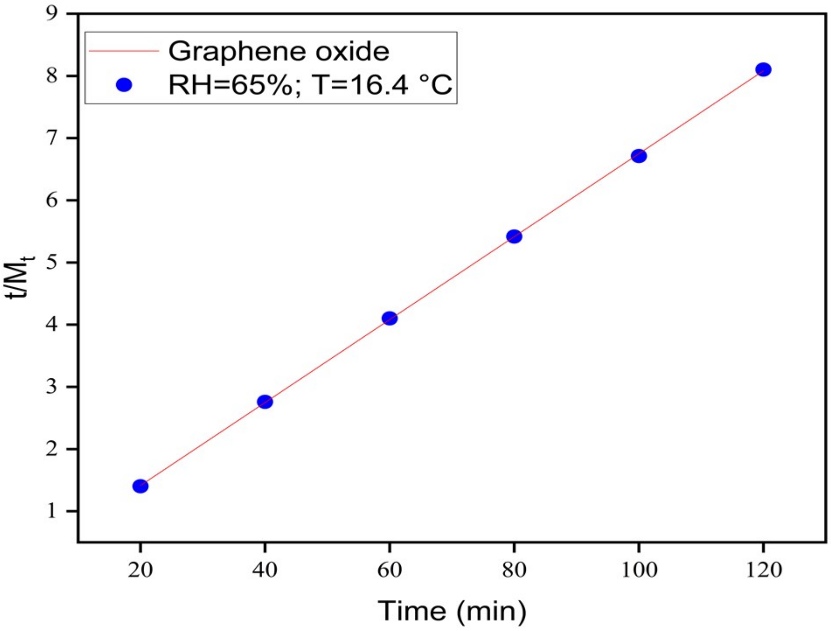

Figure S13: Pseudo 2nd order kinetics model for synthesized GO at a RH of 65 %

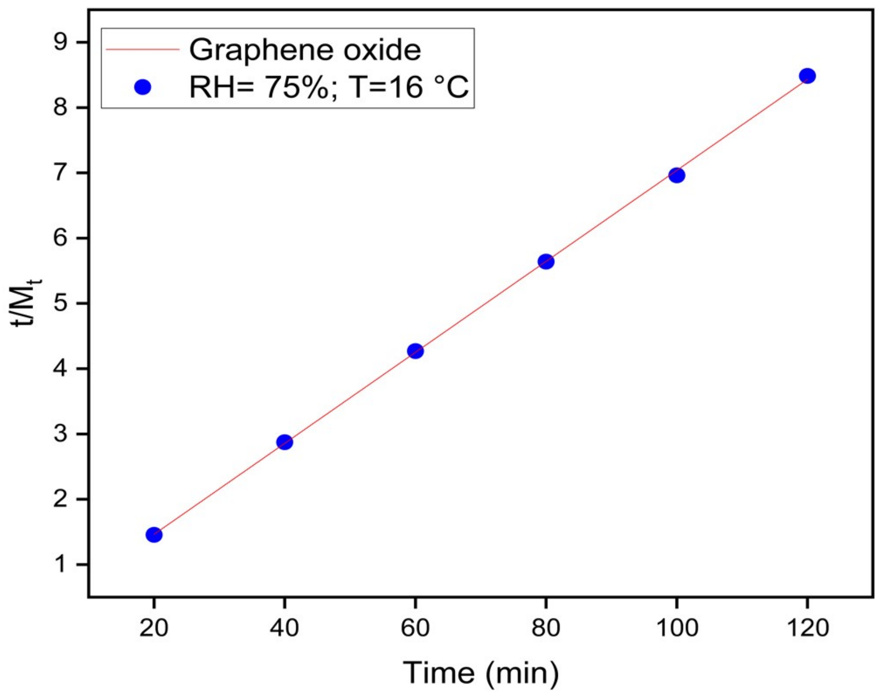

Figure S14: Pseudo 2nd order kinetics model for synthesized GO at a RH of 75 %

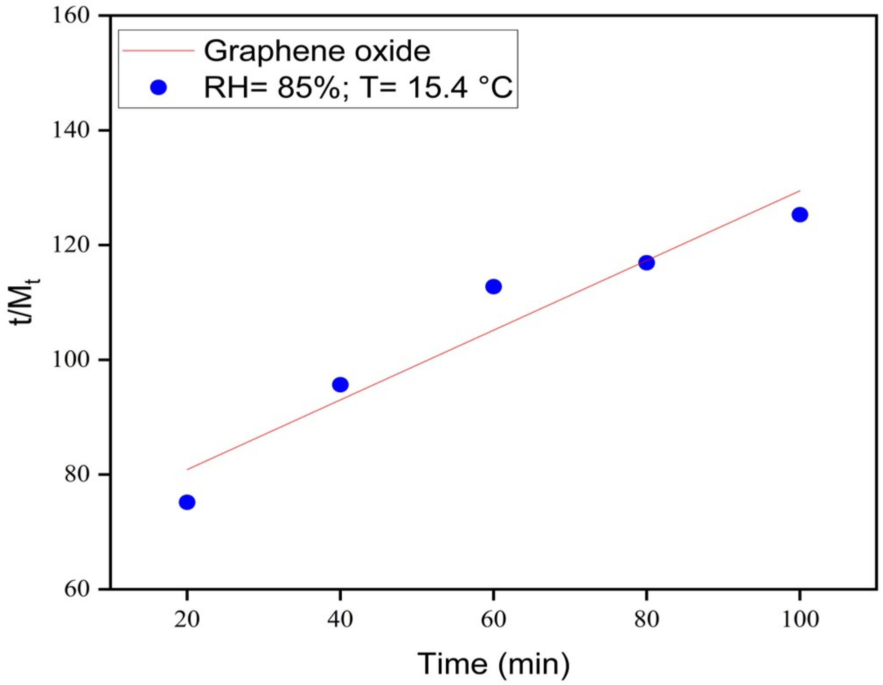

Figure S15: Pseudo 2nd order kinetics model for synthesized GO at a RH of 85 %

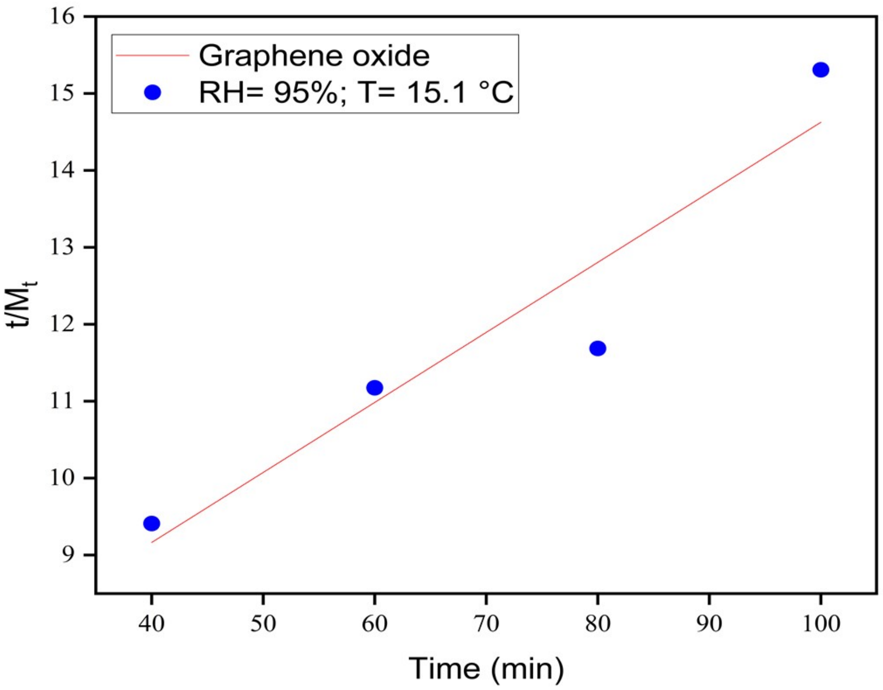

Figure S16: Pseudo 2nd order kinetics model for synthesized GO at a RH of 95 %

MOF-5:

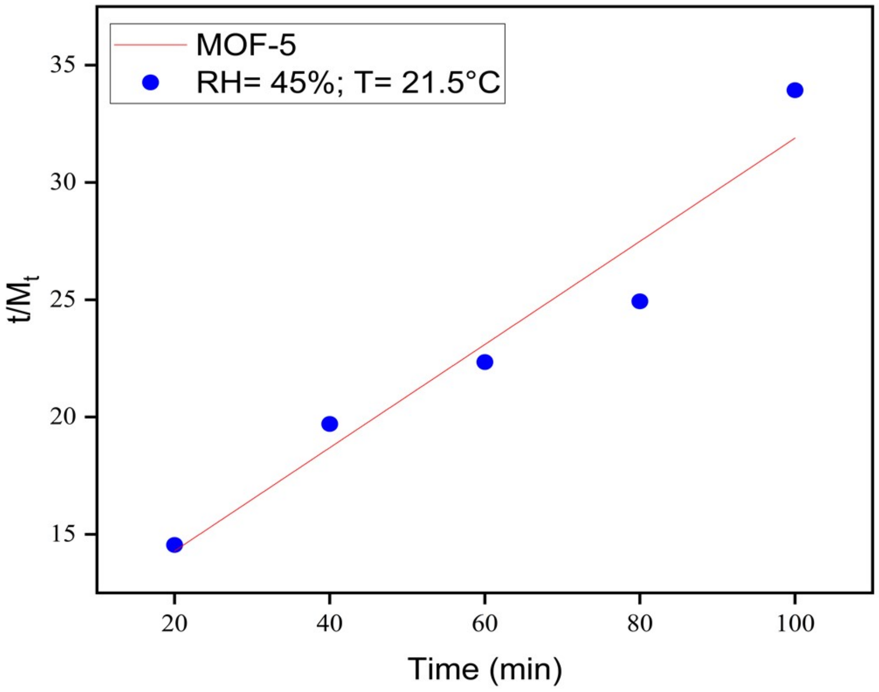

Figure S17: Pseudo 2nd order kinetics model for synthesized MOF-5 at a RH of 45 %

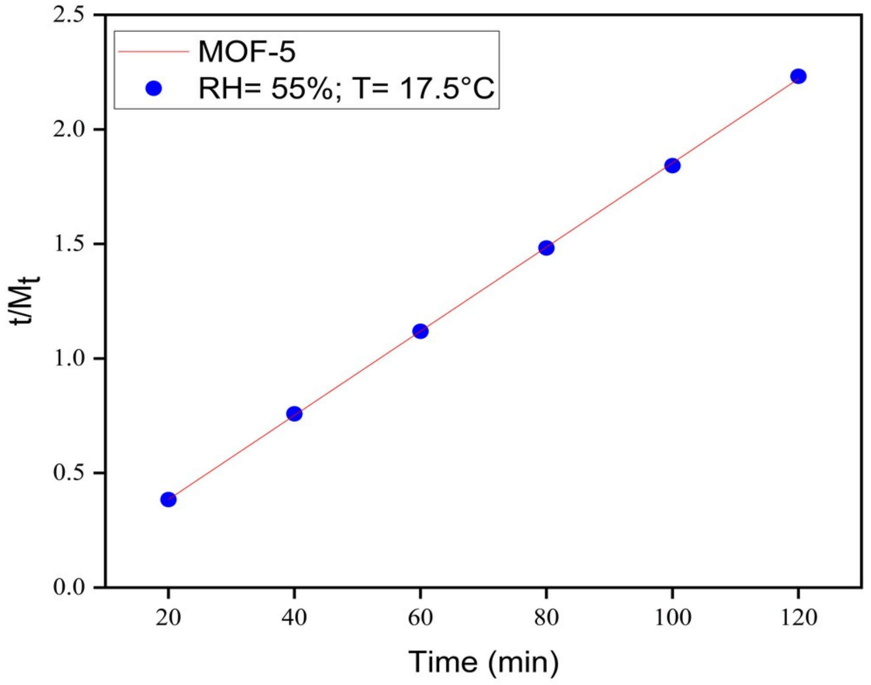

Figure S18: Pseudo 2nd order kinetics model for synthesized MOF-5 at a RH of 55 %

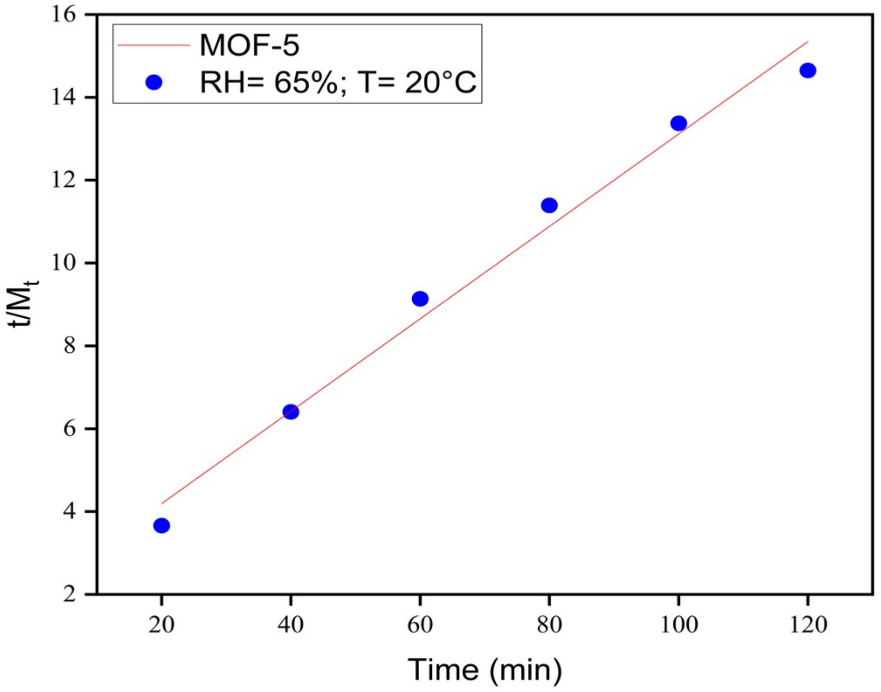

Figure S19: Pseudo 2nd order kinetics model for synthesized MOF-5 at a RH of 65 %

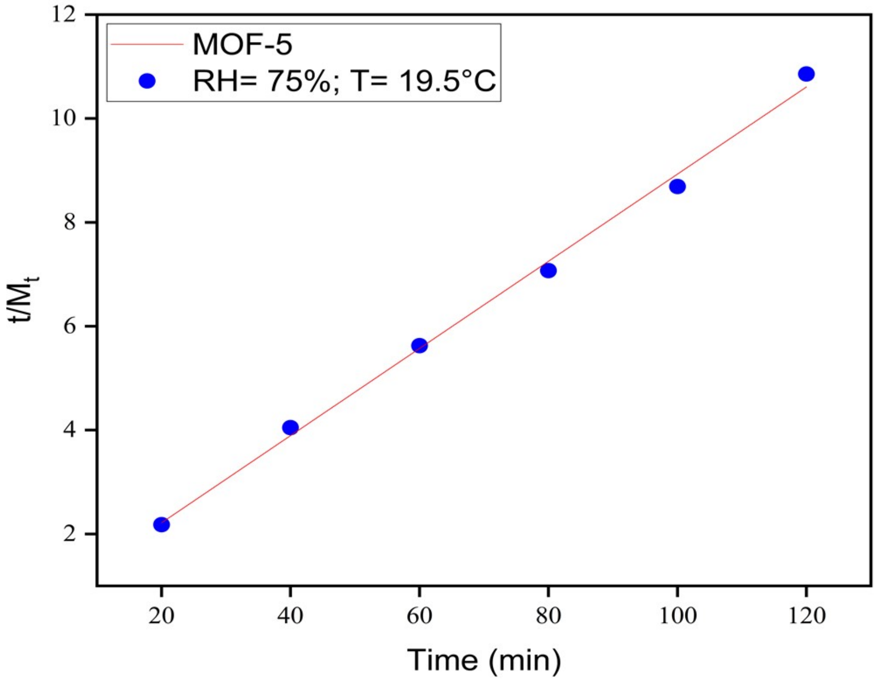

Figure S20: Pseudo 2nd order kinetics model for synthesized MOF-5 at a RH of 75 %

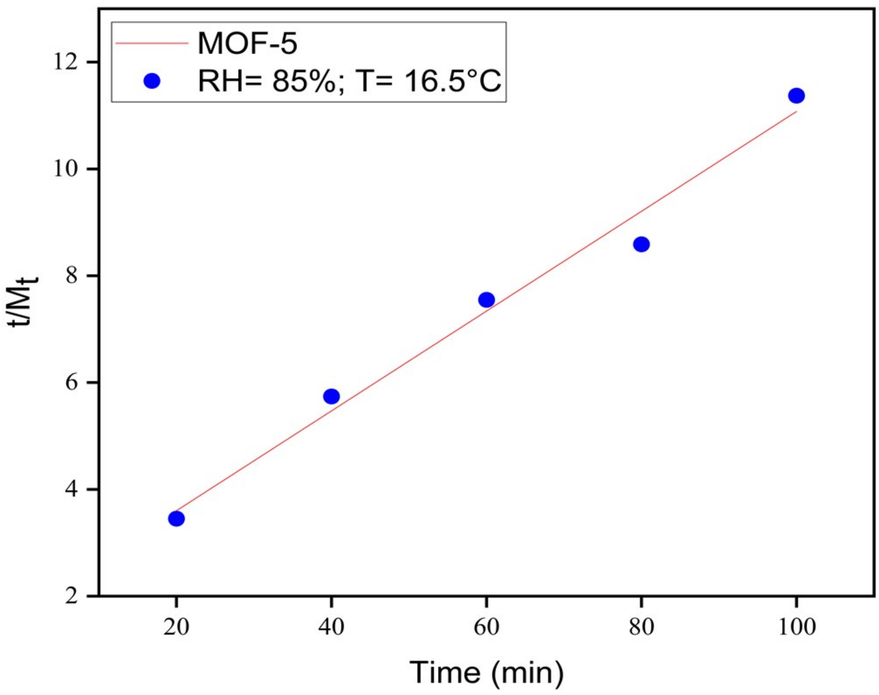

Figure S21: Pseudo 2nd order kinetics model for synthesized MOF-5 at a RH of 85 %

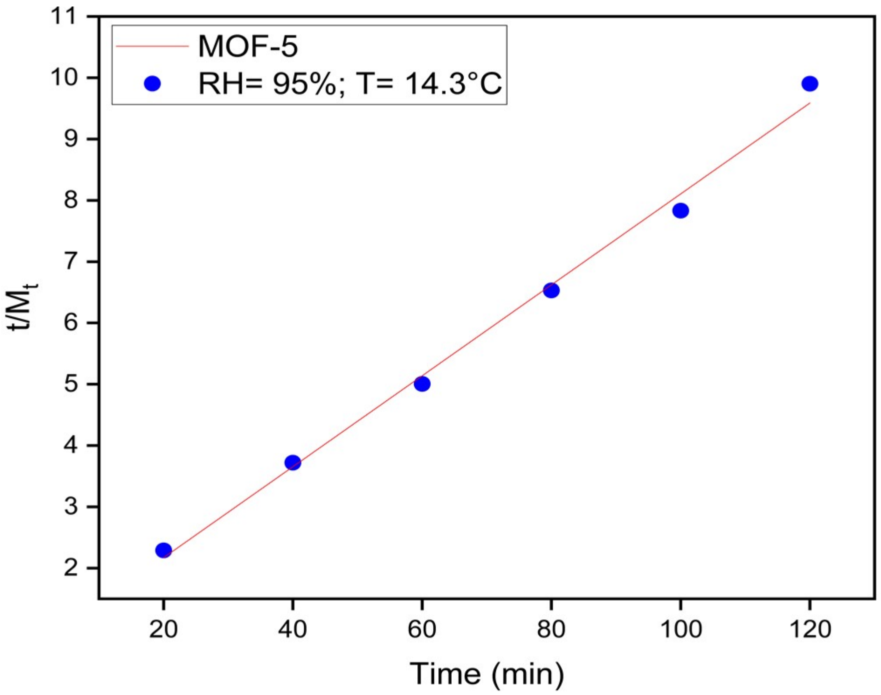

Figure S22: Pseudo 2nd order kinetics model for synthesized MOF-5 at a RH of 95 %

MOF-5/ GO composite:

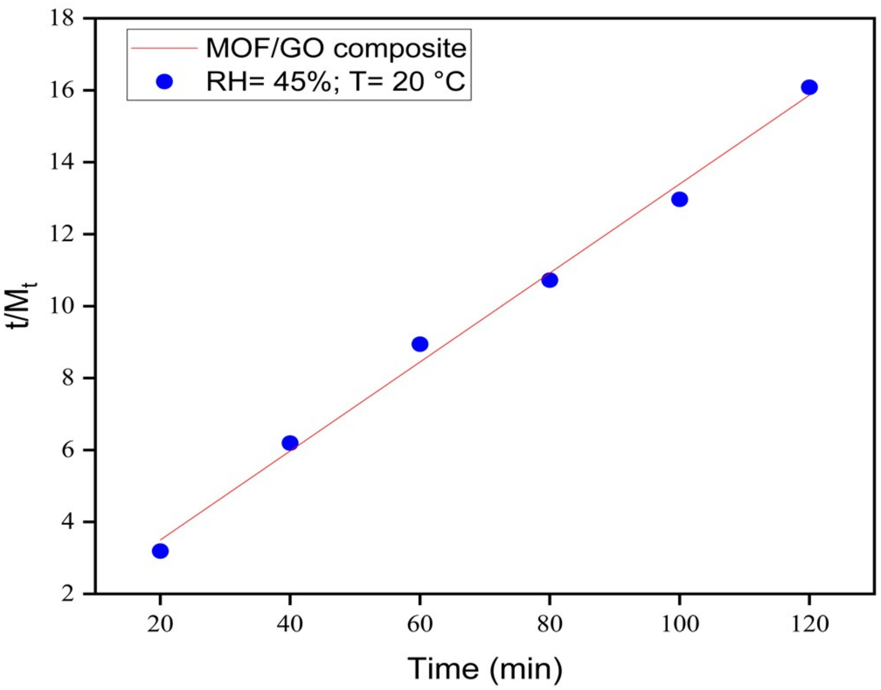

Figure S23: Pseudo 2nd order kinetics model for synthesized GO/MOF-5 composite at a RH of 45 %

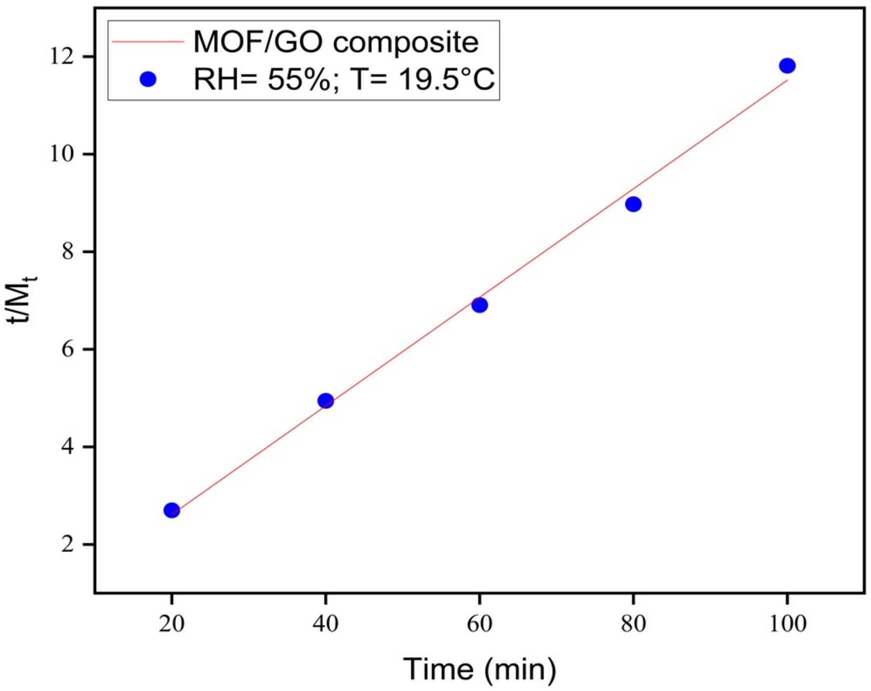

Figure S24: Pseudo 2nd order kinetics model for synthesized GO/MOF-5 composite at a RH of 55 %

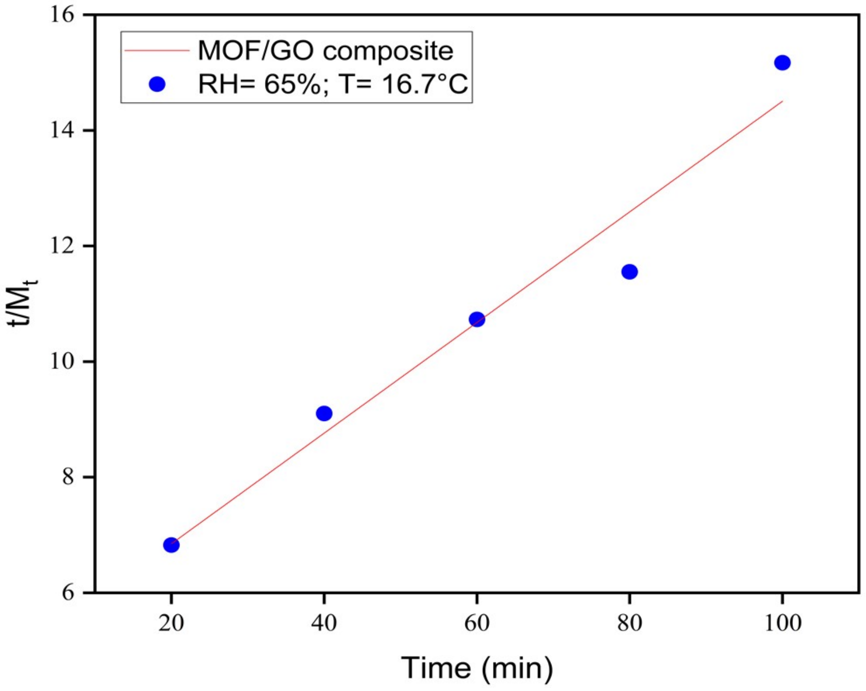

Figure S25: Pseudo 2nd order kinetics model for synthesized GO/MOF-5 composite at a RH of 65 %

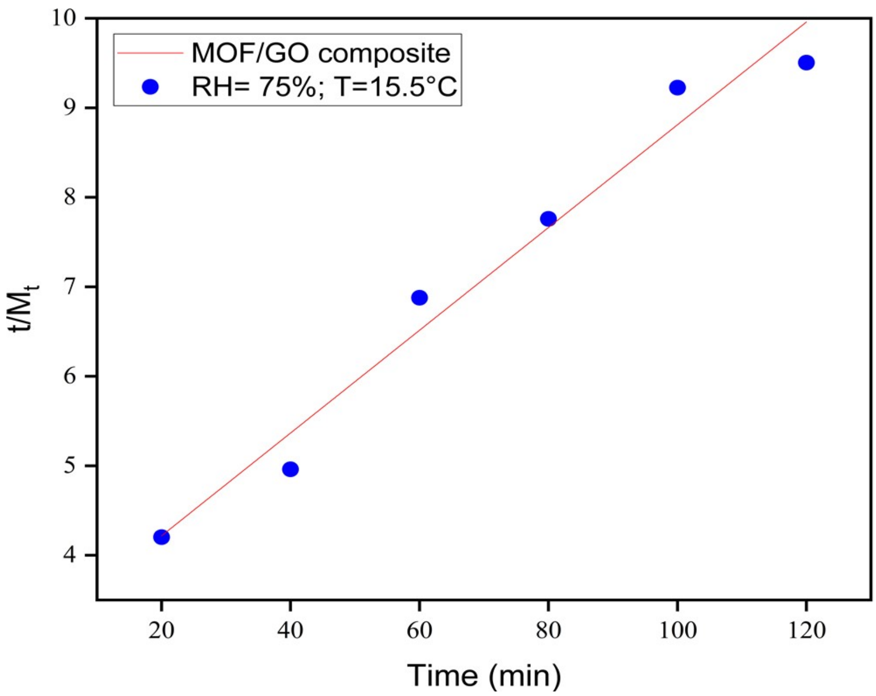

Figure S26: Pseudo 2nd order kinetics model for synthesized GO/MOF-5 composite at a RH of 75 %

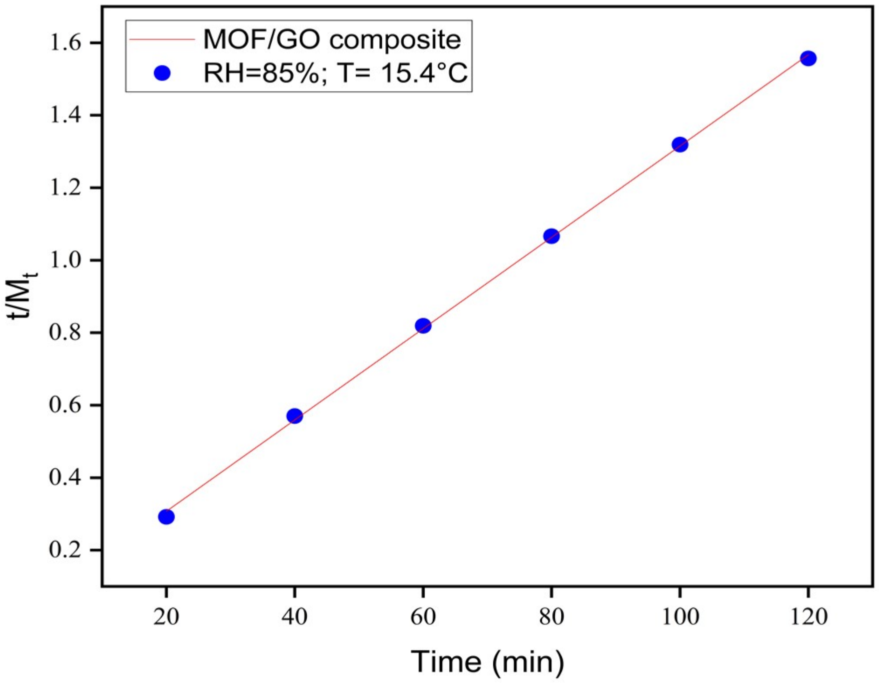

Figure S27: Pseudo 2nd order kinetics model for synthesized GO/MOF-5 composite at a RH of 85 %

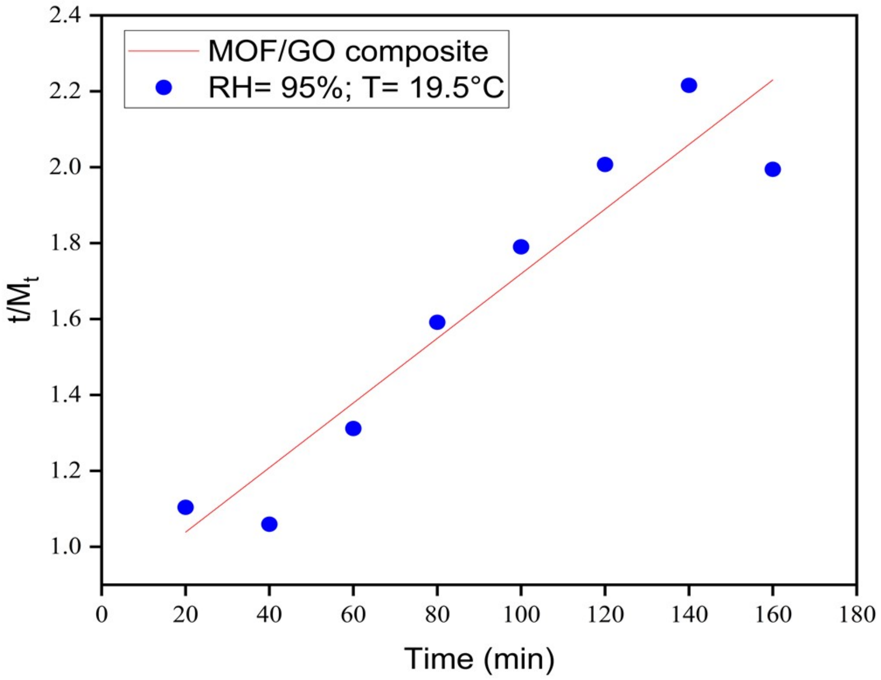

Figure S28: Pseudo 2nd order kinetics model for synthesized GO/MOF-5 composite at a RH of 95 %

Table S1: Comparison between Saturation point (Sp) and Moisture content (Mc) at different RH%

**Relative Humidity (%)**	**45 ± 2**	**55 ± 2**	**65 ± 2**	**75 ± 2**	**85 ± 2**	**95 ± 2**
**Material**	**S_p/M_c**	**S_p/M_c**	**S_p/M_c**	**S_p/M_c**	**S_p/M_c**	**S_p/M_c**
**GO**	80/56	80/53	100/148	80/141	100/8	80/60
**MOF-5**	80/32	100/542	120/80	100/115	80/93	100/127
**GO/MOF-5 composite**	100/77	80/89	80/69	140/1137	120/770	160/802

Table S2: Parameters for Pseudo 2nd order kinetics model of synthesized GO at different RH%

**Humidity (%)**	**Slope (m)**	**Intercept (c)**	**M_e (Th)**	**M_e (Exp) (mg/g)**	**k_2 (min^{-1})**

45 ± 3	0.13793	3.79648	5.75204	7.2500	0.0050
55 ± 3	0.18609	0.40871	5.32452	5.3737	0.0847
65 ± 3	0.0666	0.0808	14.8993	14.9927	0.0550
75 ± 3	0.0697	0.0695	14.3624	14.3472	0.0698
85 ± 3	1.0054	50.1459	0.79818	0.9946	0.0201
95 ± 3	0.0910	5.5234	6.84564	10.9865	0.0014

Table S3: Parameters for Pseudo 2nd order kinetics model of synthesized MOF-5 at different RH%

**Humidity (%)**	**Slope (m)**	**Intercept (c)**	**M_e (Th)**	**M_e (Exp) (mg/g)**	**k_2 (min^{-1})**
45 ± 3	4.53720	9.88877	100	4.5372	0.0049
55 ± 3	0.01836	0.0176	54.2913	54.4662	0.1915
65 ± 3	0.1115	1.9620	8.19246	8.9653	0.0634
75 ± 3	0.08392	0.53766	11.5086	11.9161	0.1309
85 ± 3	0.09345	1.73166	9.31596	10.7009	0.0050
95 ± 3	0.07417	0.6888	12.7687	1.3482	0.7987

Table S4: Parameters for Pseudo 2nd order kinetics model of synthesized GO/MOF-5 composite at different RH%

**Humidity (%)**	**Slope (m)**	**Intercept (c)**	**M_e (Th)**	**M_e (Exp) (mg/g)**	**k_2 (min^{-1})**
45 ± 3	0.12367	1.02683	7.7116	8.0860	0.01488
55 ± 3	0.11132	0.38769	8.91386	8.9831	0.03196
65 ± 3	0.0957	4.93498	6.9241	10.4993	0.01856
75 ± 3	0.05742	3.06936	13.7475	17.4155	0.01074
85 ± 3	0.0126	0.05532	77.0654	79.3651	0.02869
95 ± 3	0.00851	0.86554	80.2264	117.5088	8.338×10^{-5}
